# The Transient Receptor Potential Vanilloid 2 (TRPV2) Channel Facilitates Virus Infection Through the Ca^2+^‐LRMDA Axis in Myeloid Cells

**DOI:** 10.1002/advs.202202857

**Published:** 2022-10-19

**Authors:** Yu‐Yao Guo, Yue Gao, Yu‐Ru Hu, Yuhan Zhao, Dexiang Jiang, Yulin Wang, Youjing Zhang, Hu Gan, Chang Xie, Zheng Liu, Bo Zhong, Zhi‐Dong Zhang, Jing Yao

**Affiliations:** ^1^ Department of Gastrointestinal Surgery College of Life Sciences Zhongnan Hospital of Wuhan University Wuhan University Wuhan 430071 China; ^2^ Department of Immunology Medical Research Institute and Frontier Science Center for Immunology and Metabolism Wuhan University Wuhan 430071 China; ^3^ Wuhan Research Center for Infectious Diseases and Cancer Chinese Academy of Medical Sciences Wuhan 430071 China; ^4^ State Key Laboratory of Virology Hubei Key Laboratory of Cell Homeostasis College of Life Sciences Frontier Science Center for Immunology and Metabolism Wuhan University Wuhan 430072 China; ^5^ The Institute for Advanced Studies Wuhan University Wuhan 430072 China

**Keywords:** antiviral immunity, Ca^2+^ influx, Lrmda, myeloid cells, TRPV2, virus penetration

## Abstract

The transient receptor potential vanilloid 2 (TRPV2) channel is a nonselective cation channel that has been implicated in multiple sensory processes in the nervous system. Here, it is shown that TRPV2 in myeloid cells facilitates virus penetration by promoting the tension and mobility of cell membrane through the Ca^2+^‐LRMDA axis. Knockout of TRPV2 in myeloid cells or inhibition of TRPV2 channel activity suppresses viral infection and protects mice from herpes simplex virus 1 (HSV‐1) and vesicular stomatitis virus (VSV) infection. Reconstitution of TRPV2 but not the Ca^2+^‐impermeable mutant TRPV2^E572Q^ into *LyZ2*‐Cre;*Trpv2* ^fl/fl^ bone marrow‐derived dendritic cells (BMDCs) restores viral infection. Mechanistically, knockout of TRPV2 in myeloid cells inhibits the tension and mobility of cell membrane and the penetration of viruses, which is restored by reconstitution of TRPV2 but not TRPV2^E572Q^. In addition, knockout of TRPV2 leads to downregulation of *Lrmda* in BMDCs and BMDMs, and knockdown of *Lrmda* significantly downregulates the mobility and tension of cell membrane and inhibits viral infections in *Trpv2* ^fl/fl^ but not *LyZ2*‐Cre;*Trpv2* ^fl/fl^ BMDCs. Consistently, complement of LRMDA into *LyZ2*‐Cre;*Trpv2* ^fl/fl^ BMDCs partially restores the tension and mobility of cell membrane and promotes viral penetration and infection. These findings characterize a previously unknown function of myeloid TRPV2 in facilitating viral infection though the Ca2^+^‐LRMDA axis.

## Graphical abstract

The transient receptor potential vanilloid 2 (TRPV2) channel is a multimodal ion channel widely distributed in nervous and non‐nervous system. Here, it is shown that TRPV2 facilitates viral entry in myeloid cells via maintaining the tensile force and mobility of cell membrane dependently on the Ca^2+^‐LRMDA axis. These findings highlight essential roles of TRPV2 in viral infection in myeloid cells.

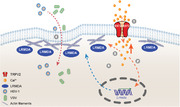

## 1 Introduction

Viruses are submicroscopic intracellular parasites that infect and replicate in host cells and viral infections can cause life‐threatening diseases to humans and animals. Characterizing the key molecular mechanisms of viral invading host cells would provide insights for therapeutic interventions of virus‐caused diseases. The successful entry of viruses into host cells constitutes the first step of an effective infection, including the attachment to plasma membrane and the subsequent penetration through the membrane into cytosol.^[^ ^1^ ^]^ The stable attachment of viruses to plasma membrane is dependent on the binding of viral particles to specific receptors on the cell surface,^[^ ^1^ ^]^ whereas the penetration of viruses is mediated through the non‐endocytic (the fusion of viral envelopes and plasma membrane) and endocytic pathways (endocytosis, caveolae, lipid raft, and macropinocytosis).^[^ ^2^ ^]^ To date, it has been well recognized that although the plasma membrane serves as a barrier for viral entry, the viral particles hijack the cell membrane machinery for their entry process. For example, the binding of simian virus 40 (SV40) to the plasma membrane induces the local activation of tyrosine kinases, leading to the rearrangement of cytoskeleton underneath the membrane for the internalization of cell membrane.^[^ ^3^, ^4^ ^]^ In addition, the interaction of herpes simplex virus 1 (HSV‐1) with the plasma membrane induces activation of receptor tyrosine kinases or integrins and dynamics of actin filaments, leading to cell membrane ruffling to form filopodia and lamellipodia.^[^ ^5^ ^]^ Consequently, the membrane‐attached viral particles move into cells along the filopodia or by fusion with the cell membrane that involves dynamics of the cell membrane controlled by the actin filament bundle. Therefore, the ordered movement of cell membrane by the cytoskeleton‐exerted mechanical force is essential for the entry of viruses.

The calcium ion (Ca^2+^) is a versatile second messenger involved in various cellular processes including signaling transduction, gene transcription, and synaptic plasticity.^[^ ^6^ ^]^ Viral infections trigger ion channels‐mediated cytosolic Ca^2+^ elevation in a cell‐type and virus‐type specific manner that benefits the entry of viruses.^[^ ^7^ ^]^ For example, the attachment of Influenza A virus (IAV) to cell surface triggers intracellular Ca^2+^ oscillations in Cos‐1, MDCK, and A549 cells through the voltage‐dependent Ca^2+^ channel (Ca_v_1.2).^[^ ^7^ ^]^ The small GTPase RhoA functions downstream of IAV‐induced Ca^2+^ oscillations to trigger the internalization of cell membrane for IAV endocytosis.^[^ ^7^ ^]^ Recent studies have demonstrated that the entry of IAV substantially induces the cytoskeletal remodeling and the formation of filopodia,^[^ ^8^ ^]^ indicating that the immediate Ca^2+^ influx triggered by viral infection induces cytoskeleton‐dependent cell membrane dynamics for the entry of viruses. Accordingly, the entry of IAV is substantially hampered by Ca^2+^ channel blockers (CCBs) or by knockdown of Ca_v_1.2 in various cell lines,^[^ ^9^ ^]^ indicating that blocking Ca^2+^ influx by targeting Ca^2+^ channels would provide plausible therapeutic strategies for virus‐related diseases. In such a context, retrospective clinical studies have shown that hospitalized patients infected with COVID‐19 or SFTSV (severe fever with thrombocytopenia syndrome virus) treated with CCB therapies exhibit reduced fatality rate.^[^ ^10^, ^11^ ^]^ It is thus of great interest to identify the ion channels that mediate virus‐triggered Ca^2+^ influx and serve as potential targets for the treatment of virus‐caused diseases.

While these previous findings have provided strong support for the critical roles of virus‐induced Ca^2+^ influx in viral entry, the roles and mechanisms of ion channels‐mediated homeostatic Ca^2+^ in viral entry are not clearly understood. The entry of vesicular stomatitis virus (VSV) is dependent on the internalization of cell membrane by the de novo clathrin‐coated pits, which involves the intracellular Ca^2+^ and the cytoskeleton underneath the cell membrane.^[^ ^12^, ^13^ ^]^ However, available studies have shown that VSV infection does not trigger Ca^2+^ influx in mouse embryonic fibroblasts (MEFs) or baby hamster kidney (BHK) cells,^[^ ^14^ ^]^ and that treatment with CCBs has no effect on VSV infection.^[^ ^15^ ^]^ It remains to be determined whether and how other Ca^2+^ permeable channels regulate the homeostatic Ca^2+^ and thereby facilitate the entry of VSV.

The transient receptor potential vanilloid 2 (TRPV2) channel is a calcium‐permeable cation channel belonging to the TRP superfamily. As a multimodal ion channel, TRPV2 channel is widely distributed in nervous and non‐nervous system, and has been implicated in diverse biological functions including thermal sensation,^[^ ^16^ ^]^ osmotic‐ or mechanosensation,^[^ ^17^, ^18^ ^]^ neuronal development,^[^ ^19^ ^]^ physiological cardiac structure and maintenance and function,^[^ ^20^ ^]^ insulin secretion,^[^ ^21^ ^]^ oncogenesis,^[^ ^22^ ^]^ and proinflammatory process.^[^ ^23^, ^24^ ^]^ TRPV2 has also been found to be expressed in immune cells and regulates various immune responses.^[^ ^25^ ^]^ For example, TRPV2 is found in macrophages to promote lipopolysaccharide‐induced tumor necrosis factor and interleukin‐6 production.^[^ ^26^ ^]^ In mast cells, TRPV2 induces Ca^2+^ influx and proinflammatory degranulation in response to mechanical and heat stimulation.^[^ ^27^ ^]^ In addition, TRPV2 is found in the immune synapse during antigen presentation to orchestrate Ca^2+^ signal in T cell activation, proliferation, and effector functions.^[^ ^28^ ^]^ Finally, TRPV2 is expressed in CD19^+^ B lymphocytes where it regulates Ca^2+^ release during B cell development and activation.^[^ ^25^ ^]^ Our transcriptomic data of bone marrow‐derived dendritic cells (BMDCs) and macrophages (BMDMs) suggest that the *Trpv2* mRNA exhibited highest expression among the *Trpv* families in these cells (Figure S1A,B and Table S1, Supporting Information). However, whether and how TRPV2 modulates homeostatic and virus‐induced Ca^2+^ signal in myeloid cells to regulate viral infections are completely unclear.

Here we show that TRPV2 facilitates virus penetration through the Ca2^+^‐LRMDA axis in myeloid cells by regulating the tension and mobility of cell membrane. We found that knockout of TRPV2 in myeloid cells significantly inhibited the penetration but not the attachment of multiple viruses including HSV‐1, VSV, IAV, and SV40 and that the *Lyz2*‐Cre;*Trpv2* ^fl/fl^ mice were more resistant to lethal HSV‐1 and VSV infection than the *Trpv2* ^fl/fl^ mice. In addition, we have determined that reconstitution of wild‐type TRPV2 or TRPV1 but not TRPV2^E572Q^ and TRPV1^D646N/E648/651Q^ (which lost the Ca^2+^ permeability) or TRPV2^E594/604Q^ (which lost the channel activity) into TRPV2‐deficent BMDCs restored the penetration of viruses, indicating that TRPV channels‐mediated Ca^2+^ permeability controls viral entry in myeloid cells. Consistently, mice treated with the TRPV2 inhibitor SKF96365 exhibited hyper‐resistance to HSV‐1 and VSV infections compared to mice treated with dimethyl sulfoxide (DMSO). Mechanistically, the homeostatic Ca^2+^ permeability of TRPV2 promoted the mechanic force for cell membrane tension and mobility through the upregulation of LRMDA. Moreover, knockdown of LRMDA in TRPV2‐sufficient BMDCs impaired the cell membrane tension and viral penetration, whereas complementation of LRMDA into TRPV2‐deficent cells substantially restored cell membrane tension and viral penetration. Thus, these findings highlight TRPV2 as a positive regulator for viral entry in myeloid cells and provide new potential target for treatment of virus‐caused diseases.

## 2 Results

### 2.1 Functional TRPV2 is Intensively Expressed in BMDCs and BMDMs

The transient receptor potential (TRP) channels are nonselective cation channels that are grouped into seven subfamilies, including TRPA, TRPC, TRPM, TRPN, TRPML, TRPP, and TRPV.^[^ ^29^ ^]^ TRPV2 belongs to the TRPV subfamily and is involved in various sensing progresses.^[^ ^30^ ^]^ We found that *Trpv2* mRNA exhibited highest expression among the *Trpv* family members in BMDMs and BMDCs(Figure S1A,B and Table S1, Supporting Information). As illustrated in Figure S1C (Supporting Information), we also detected a high abundance of expression of TRPV2 channel protein in BMDMs and BMDCs via immunoblotting. We then used patch‐clamp technique to examine the activity of TRPV2 channel distributed in BMDCs and BMDMs. Notably, whole‐cell recordings at a holding potential of −60 mV in BMDCs and BMDMs showed that 2‐Aminoethyl diphenylborinate (2‐APB) evoked a similar response to that in HEK293 cells transiently transfected with TRPV2 (Figure S1D, Supporting Information). The dose‐response curves to 2‐APB were fitted with a Hill equation, and the corresponding EC_50_ values and Hill coefficients were assessed to be all similar (Figure S1E, Supporting Information), i.e., EC_50_ = 2.26 ± 0.03 mm and *n*_H_ = 5.39 ± 0.31 for BMDCs (*n* = 5); EC_50_ = 2.41 ± 0.04 mm and *n*_H_ = 5.37 ± 0.37 for BMDMs (*n* = 5); and EC_50_ = 2.26 ± 0.03 mM and *n*_H_ = 5.39 ± 0.31 for HEK293‐TRPV2 (*n* = 5), respectively. Additionally, the 2‐APB‐evoked currents in BMDMs and BMDCs were effectively inhibited by SKF96365, an inhibitor of TRPV2 channel, in a dose‐dependent manner (Figure S1F,G, Supporting Information), similar to the observation in TRPV2‐expressing HEK293 cells. As TRPV2 is highly permeable to Ca^2+^, we next compared the activation of TRPV2 by cannabidiol (CBD) using a calcium imaging assay with GCaMP6m in BMDCs, BMDMs, and TRPV2‐expressing HEK293 cells. We observed that CBD (30 µM) evoked robust Ca^2+^ increase in all three different types of cells (Figure S1H,I, Supporting Information). Ionomycin (1 µm) was subsequently applied to ascertain cell viability at the end of each experiment. Together, these results suggest that TRPV2 is intensively expressed in BMDCs and BMDMs and exerts good ion channel activity.

### 2.2 TRPV2 is Dispensable for Immune Cell Differentiation and Development

**Figure 1:**
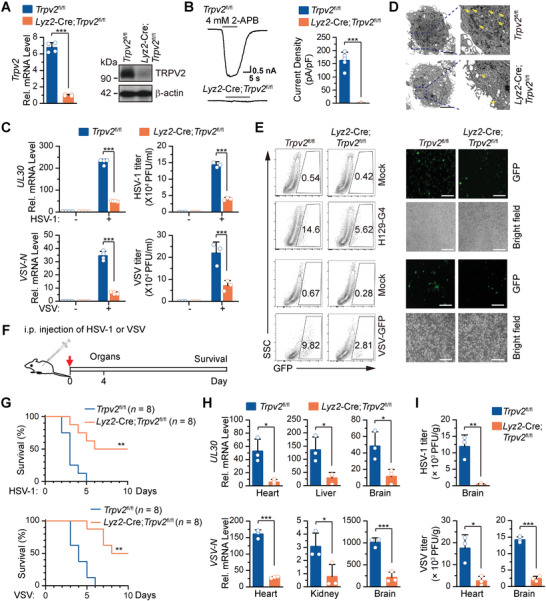
Knockout of TRPV2 protects mice from lethal HSV‐1 and VSV infection. A) qRT‐PCR and Immunoblot analysis of *Trpv2* mRNA (left) and TRPV2 protein (right) in *Trpv2* ^fl/fl^ and *Lyz2‐*Cre;*Trpv2* ^fl/fl^ BMDCs. B) A representative whole‐cell recording of the *Trpv2* ^fl/fl^ (left, upper) and *Lyz2*‐Cre;*Trpv2* ^fl/fl^ (left, lower) BMDCs. The cell was exposed to 4 mm 2‐APB in neutral condition (pH 7.4). Summary data (right graph) of current densities evoked by 4 m M 2‐APB at a holding potential of −60 mV. C) qRT‐PCR analysis of HSV‐1 *UL30* gene or VSV *N* gene in (left) and HSV‐1 and VSV titers in the supernatants of (right) *Trpv2* ^fl/fl^ and *Lyz2*‐Cre;*Trpv2* ^fl/fl^ BMDCs infected with HSV‐1 or VSV for 12 h. D) Transmission electron microscopy analysis of *Trpv2* ^fl/fl^ and *Lyz2*‐Cre;*Trpv2* ^fl/fl^ BMDCs that were infected with HSV‐1 for 12 h. E) Flow cytometry analysis (left) and fluorescent microscopy imaging (right) of *Trpv2* ^fl/fl^ and *Lyz2*‐Cre;*Trpv2* ^fl/fl^ BMDCs that were left uninfected or infected with H129‐G4 or VSV‐GFP for 1 h followed by PBS wash twice and cultured in full medium for 12 h. Numbers adjacent to the outlined areas indicate percentages of GFP^+^ cells. F) A scheme of viral infection and analysis of *Trpv2* ^fl/fl^ and *Lyz2*‐Cre;*Trpv2* ^fl/fl^ mice. G) Survival (Kaplan–Meier curve) of *Trpv2* ^fl/fl^ (*n*  =  8) and *Lyz2*‐Cre;*Trpv2* ^fl/fl^ (*n*  =  8) mice that were intraperitoneally injected with HSV‐1 (upper, 5 × 10^6^ PFU per mouse) or VSV (lower, 2 × 10^7^ PFU per mouse) and monitored for 10 days. H) qRT‐PCR analysis of HSV‐1 *UL30* gene (upper) or VSV *N* gene (lower) in the heart, liver, brain, or kidney of *Trpv2* ^fl/fl^ (*n*  =  3) and *Lyz2*‐Cre;*Trpv2* ^fl/fl^ mice (*n*  =  3) that were intraperitoneally injected with HSV‐1 (2.5 × 10^6^ PFU per mouse) or VSV (1 × 10^7^ PFU) for 4 days. I) Plaque assays analyzing HSV‐1 (upper) in the brain or VSV (lower) titers in the brain and the heart from *Trpv2* ^fl/fl^ (*n*  =  3) and *Lyz2*‐Cre;*Trpv2* ^fl/fl^ (*n*  =  3) mice that were intraperitoneally injected with HSV‐1 (2.5 × 10^6^ PFU per mouse) or VSV (1 × 10^7^ PFU) for 4 days. *p < 0.05; **p < 0.01; ***p < 0.001 (two‐tailed student's *t*‐test in A–C,H–I, and Log‐Rank analysis in G). Graphs show mean ± S.D. in A–C,G–I). Scale bars represent 200 µm in E). Data are combined two G) independent experiments or representative of two A–E,H–I) independent experiments.

To examine the functions of TRPV2 in myeloid cells in vivo, we generated *Trpv2* ^fl/+^ mice through CRISPR/Cas9‐mediated gene editing (Figure S2A, Supporting Information). Southern blot analysis suggested the targeting vector was successfully recombined with the wild‐type *Trpv2* allele (Figure S2B, Supporting Information). Cre recombinase‐mediated deletion of exon 4 and exon 5 with flanking loxp sites would result in an early translational termination of TRPV2(aa1‐270) or *Trpv2* mRNA instability (Figure S2C,D, Supporting Information). We obtained *Lyz2*‐Cre*;Trpv2* ^fl/fl^ mice by crossing the *Trpv2* ^fl/+^ mice with *Lyz2*‐Cre mice and found that *Lyz2*‐Cre;*Trpv2* ^fl/fl^ mice bred and developed normally as did the *Trpv2* ^fl/fl^ mice. Results from quantitative RT‐PCR and immunoblot analysis showed that TRPV2 was almost completely depleted in *Lyz2*‐Cre;*Trpv2* ^fl/fl^ BMDCs and BMDMs (**Figure** **1**; and Figure S2E, Supporting Information). In addition, the *Lyz2*‐Cre;*Trpv2* ^fl/fl^ BMDCs and BMDMs failed to respond to 2‐APB stimulation (Figure 1B; and Figure S2F, Supporting Information), suggesting that the strategy to knockout TRPV2 in mice is reliable and successful.

We further observed that knockout of TRPV2 in myeloid cells did not affect the differentiation of BMDMs, BMDCs, or Flt3L‐cDCs in in vitro cultures with M‐CSF, GM‐CSF, or Flt3L, respectively (Figure S2G, Supporting Information). In addition, the percentages and numbers of myeloid CD11b^+^ and CD11c^+^ populations or lymphoid CD4^+^, CD8^+^, or CD19^+^ populations in spleen, inguinal lymph nodes and peripheral blood from *Lyz2*‐Cre;*Trpv2* ^fl/fl^ mice were similar to those from *Trpv2* ^fl/fl^ mice (Figure S3A–D, Supporting Information). These data indicate that TRPV2 is dispensable for the differentiation and development of immune cells.

### 2.3 Knockout of TRPV2 in Myeloid‐Derived Cells Leads to Resistance to Viral Infection

Considering the essential roles of myeloid cells in defense against viral infection, we next examined whether depletion of TRPV2 in myeloid cells affected cellular antiviral responses. The *Lyz2*‐Cre;Trpv2^fl/fl^ BMDCs and BMDMs and the Trpv2^fl/fl^ counterparts were infected with HSV‐1 or VSV. One hour later, the cells were washed with phosphate buffered saline (PBS) and cultured in medium for 12 h followed by various experiments. Results from qRT‐PCR analysis suggested that the mRNA levels of HSV‐1 *UL30* gene and VSV *N* gene were significantly lower in *Lyz2*‐Cre;*Trpv2* ^fl/fl^ BMDCs and BMDMs than in the *Trpv2* ^fl/fl^ counterparts (Figure 1C; and Figure S4A, Supporting Information). The virus titers in the supernatants of and the viral particles inside the *Lyz2*‐Cre;*Trpv2* ^fl/fl^ BMDCs or BMDMs were significantly decreased compared to the *Trpv2* ^fl/fl^ counterparts as revealed by plaque assays and electron microscopy analysis (Figure 1C,D; and Figure S4A, Supporting Information). Consistently, the replication of H129‐G4 (a GFP‐tagged strain of HSV‐1) or VSV‐GFP was substantially inhibited in the *Lyz2*‐Cre;*Trpv2* ^fl/fl^ BMDCs and BMDMs compared to the *Trpv2* ^fl/fl^ counterparts as monitored by flow cytometry analysis and fluorescent imaging of the GFP signals (Figure 1E; and Figure S4B, Supporting Information). These data together suggest that depletion of TRPV2 in myeloid cells inhibits viral infections.

We next examined the role of myeloid TRPV2 in host defense against viruses in mice. The *Lyz2*‐Cre;*Trpv2* ^fl/fl^ mice and the *Trpv2* ^fl/fl^ mice were intraperitoneally injected with of HSV‐1 or VSV followed by various analysis (Figure 1F). Interestingly, the *Lyz2*‐Cre;*Trpv2* ^fl/fl^ mice exhibited delayed death and prolonged survival compared to the Trpv2^fl/fl^ mice after viral infection (Figure 1G). Consistently, the levels of HSV‐1 *UL30* gene and VSV *N* gene were significantly lower in various organs such as heart and brain of the *Lyz2*‐Cre;*Trpv2* ^fl/fl^ mice than in those of the *Trpv2* ^fl/fl^ mice (Figure 1H). In addition, the HSV‐1 titers in the brain and VSV titers in the heart and brain of the *Lyz2*‐Cre;*Trpv2* ^fl/fl^ mice were significantly decreased compared to the *Trpv2* ^fl/fl^ mice (Figure 1I). Collectively, these data demonstrate that knockout of TRPV2 in myeloid cells leads to resistance to viral infections in mice.

### 2.4 Inhibition of the Ion Channel Activity of TRPV2 Restricts Viral Infection

**Figure 2:**
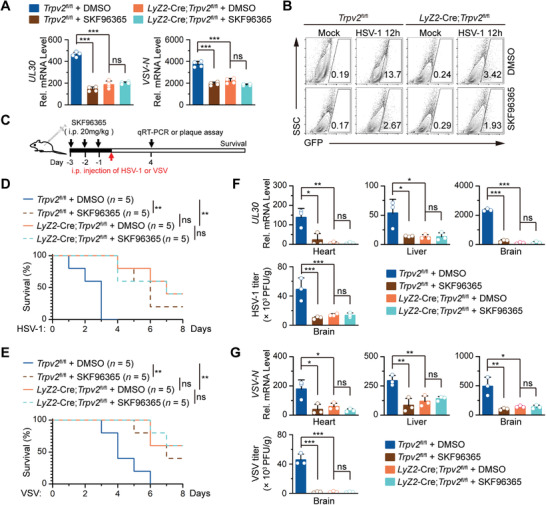
Inhibition of TRPV2 channel activity by SKF96365 inhibits viral infection. A) qRT‐PCR analysis of HSV‐1 *UL30* gene in *Trpv2* ^fl/fl^ and *Lyz2*‐Cre;*Trpv2* ^fl/fl^ BMDCs that were pretreated with DMSO or SKF96365 (100 µm) for 2 h followed by HSV‐1 infection for 12 h. B) Flow cytometry analysis of *Trpv2* ^fl/fl^ and *Lyz2*‐Cre;*Trpv2* ^fl/fl^ BMDCs that were pretreated with DMSO or SKF96365 (100 µm) for 2 h followed by H129‐G4 infection for 12 h. Numbers adjacent to the outlined areas indicate percentages of GFP^+^ cells. C) A scheme of SKF96365 treatment and viral infection of *Trpv2* ^fl/fl^ and *Lyz2*‐Cre;*Trpv2* ^fl/fl^ mice. The *Trpv2* ^fl/fl^ and Lyz2‐Cre *Trpv2* ^fl/fl^ mice were intraperitoneally injected with DMSO or SKF96365 (20 mg kg^−1^ per mouse) for 3 successive days followed by intraperitoneal injection of HSV‐1 or VSV. D–E) Survival (Kaplan–Meier curve) of *Trpv2* ^fl/fl^ (*n*  = 5) and *Lyz2*‐Cre;*Trpv2* ^fl/fl^ mice (*n* = 5) that were treated as in C) and intraperitoneally injected with either HSV‐1 (D, 5 × 10^6^ PFU per mouse) or VSV (E, 2 × 10^7^ PFU per mouse) monitored survival for 8 days. F) qRT‐PCR analysis of HSV‐1 *UL30* gene (in the heart, liver, and brain) and HSV‐1 titers (in the brain) from *Trpv2* ^fl/fl^ (*n* = 3) and Lyz2‐Cre;*Trpv2* ^fl/fl^ (*n* = 3) mice similarly treated as in D) except for that HSV‐1 (2.5 × 10^6^ PFU per mouse) was intraperitoneally injected for 4 days. G) qRT‐PCR analysis of VSV *N* gene (in the heart, liver, and brain) and VSV titers (in the brain) from *Trpv2* ^fl/fl^ (*n* = 3) and Lyz2‐Cre *Trpv2* ^fl/fl^ (*n* = 3) mice treated as in E) except for that VSV (1 × 10^7^ PFU per mouse) was intraperitoneally injected for 4 days. *p < 0.05; **p < 0.01; ***p < 0.001; ns, not significant (two‐tailed student's *t*‐test in A,F,G), and Log‐Rank analysis in D,E). Graphs show mean ± S.D. in A,F,G). Data are representative of two D,E) or three independent experiments or representative of three A,B, F,G) independent experiments.

Because SKF96365 suppresses the ion channel activity of TRPV2 in BMDCs (Figure S1F,G, Supporting Information), we further asked whether the inhibitory effect of SKF96365 on TRPV2 would affect the viral infection of BMDCs. Results from qRT‐PCR analysis suggested that treatment of SKF96365 significantly inhibited the mRNA levels of HSV‐1 *UL30* gene in Trpv2^fl/fl^ BMDCs but not in *Lyz2*‐Cre;Trpv2^fl/fl^ BMDCs (**Figure** **2**A). We also used H129‐G4 to infect the BMDCs and performed flow cytometry analysis. As shown in Figure 2B, we observed that SKF96365 treatment substantially inhibited the replication of H129‐G4 in *Trpv2* ^fl/fl^ BMDCs but not in *Lyz2*‐Cre;*Trpv2* ^fl/fl^ BMDCs as monitored by the GFP signals (Figure 2B), indicating that TRPV2 is the primary target of SKF96365 in BMDCs and that inhibition of the ion channel activity of TRPV2 by SKF96365 substantially prevents viral infection in BMDCs. These data are in agreement with our previous observation that the *Trpv* genes except for *Trpv2* are rarely expressed in BMDCs (Figure S1A,B; and Table S1, Supporting Information).

We next examined whether inhibition of TRPV2 by SKF96365 affected antiviral responses in mice. The *Trpv2* ^fl/fl^ and the *Lyz2*‐Cre;*Trpv2* ^fl/fl^ mice were intraperitoneally (i.p.) injected with SKF96365 or DMSO for 3 successive days followed by i.p. injection with HSV‐1 (5 × 10^6^ PFU per mouse) or VSV (2 × 10^7^ PFU per mouse) (Figure 2C). Injection of SKF96365 significantly delayed the death and prolonged the survival of the *Trpv2* ^fl/fl^ mice but not the *Lyz2*‐Cre;*Trpv2* ^fl/fl^ mice after viral infection (Figure 2D,E). In line with these observations, the viral titers in the brain and the expression levels of HSV‐1 *UL30* gene or VSV *N* gene in various tissues of *Trpv2* ^fl/fl^ mice but not *Lyz2*‐Cre;*Trpv2* ^fl/fl^ mice were significantly reduced by SKF96365 treatment (Figure 2F,G), indicating that myeloid TRPV2 is the primary target of SKF96365 in mice defense against viral infection and that SKF96365‐mediated inhibition of TRPV2 activity promotes antiviral responses. We next performed experiments with wild‐type C57BL/6 mice that were simultaneously injected with HSV‐1 (2.5 × 10^6^ PFU per mouse, i.p.) or VSV (1 × 10^7^ PFU per mouse, i.p.) and SKF96365 or DMSO followed by injection with SKF96365 or DMSO for three successive days. The results suggested that SKF96365 delayed the death and prolonged the survival of mice (Figure S5A,B, Supporting Information). The levels of HSV‐1 *UL30* gene or VSV *N* gene in the heart, liver and brain and the HSV‐1 or the VSV titers in brain were significantly decreased in mice injected with SKF96365 compared to those in mice injected with DMSO (Figure S5C,D, Supporting Information). Taken together, these data suggested that inhibition of the ion channel activity of TRPV2 promotes antiviral responses.

### 2.5 TRPV2 Mediates HSV‐1‐Triggered Ca^2+^ Influx in BMDMs and BMDCs

**Figure 3:**
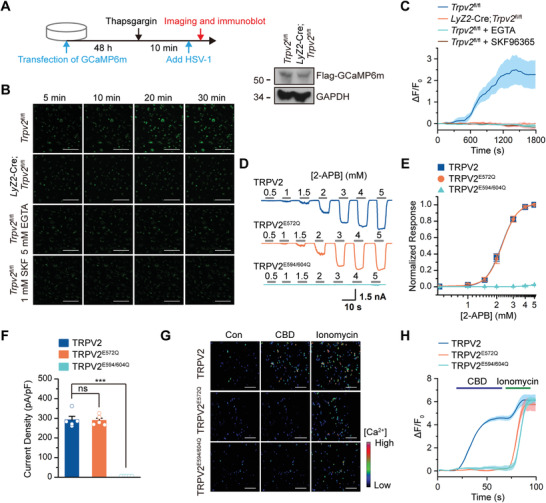
E572 of TRPV2 is required for the Ca^2+^ permeability. A) A scheme of experiments (left) and immunoblot analysis (right) of GCaMP6m in *Trpv2* ^fl/fl^ and *Lyz2*‐Cre;*Trpv2* ^fl/fl^ BMDCs for B,C). B) Fluorescent microscopy analysis of Ca^2+^ imaging in *Trpv2* ^fl/fl^ and *Lyz2*‐Cre;*Trpv2* ^fl/fl^ BMDCs infected with HSV‐1 in the presence of EGTA (5 mm) or SKF96365 (1 mm). C) Quantitative analysis of the GFP signals in *Trpv2* ^fl/fl^ and *Lyz2*‐Cre;*Trpv2* ^fl/fl^ BMDCs treated as in B). D) Representative whole‐cell recordings of HEK293 cells transfected with wild‐type TRPV2, TRPV2^E572Q^, or TRPV2^E594/604Q^ that were stimulated with 2‐APB (0.5–5 mm) under the holding potential of −60 mV. E) Dose‐response curves to 2‐APB of HEK293 cells treated as in D). Fitting by Hill's equation resulted in the following: EC_50_ = 2.25 ± 0.02 mm, *n*_H_ = 5.37 ± 0.28 for TRPV2 (*n* = 5); EC_50_ = 2.28 ± 0.02 mm, *n*_H_ = 5.47 ± 0.22 for TRPV2^E572Q^ (*n* = 5). F) Summary plot of current density. The current densities evoked by 2‐APB were determined by normalizing the membrane peak current by membrane capacitance (*n* = 5). G) Fluorescent microscopy analysis of Ca^2+^ imaging in GCaMP6m‐expression HEK293 cells transfected with TRPV2, TRPV2^E572Q^, or TRPV2^E594/604Q^ followed by treatment with DMSO (Con), CBD (30 µm), or ionomycin (1 µm). H) Averaged responses of HEK293 cells treated as in G). GCaMP6m fluorescence changes were computed as (Fi–F0)/F0, where Fi represented fluorescence intensity at any frame and F0 was the baseline fluorescence calculated from the averaged fluorescence of the first 10 frames. ***p < 0.001; ns, not significant (two‐tailed student's *t*‐test in F). Graphs show mean ± S.D. in C,E,F,H). Scale bars represent 100 µm in B) and 200 µm in G). Data are representative of three B–H) independent experiments.

Because HSV‐1 infection triggers increase of Ca^2+^ in the cytosol which facilitates viral infection and TRPV2 channel is a non‐selective ion channel and has relatively high Ca^2+^ permeability, we next examined whether viral infection increased cytosolic Ca^2+^ through TRPV2. We transfected GCaMP6m into *Lyz2*‐Cre;*Trpv2* ^fl/fl^ or *Trpv2* ^fl/fl^ BMDCs and BMDMs. Forty‐eight hours later, the cells were treated with thapsigargin (1 µm) for 10 min to induce intracellular Ca^2+^ release followed by infection with HSV‐1 in the presence of ethylene glycol tetraacetic acid (EGTA) or SKF96365 with live cell imaging (**Figure** **3**A). The results suggested that HSV‐1 infection significantly increased the cytosolic Ca^2+^ in *Trpv2* ^fl/fl^ but not *Lyz2*‐Cre;*Trpv2* ^fl/fl^ BMDCs and BMDMs within 30 min as indicated by the GFP signals (Figure 3B,C; and Figure S6A,B, Supporting Information). Treatment with EGTA or SKF96365 completely diminished the increase of Ca^2+^ in *Trpv2* ^fl/fl^ BMDCs and BMDMs triggered by HSV‐1 infection (Figure 3B,C; and Figure S6A,B, Supporting Information), indicating that HSV‐1 triggers Ca^2+^ influx in BMDCs and BMDMs in a manner dependent on TRPV2.

### 2.6 E572 is a Critical Residue for TRPV2‐Mediated the Calcium Influx

Previous studies have demonstrated that the negatively charged residues E594 and E604 in the pore region (the linker between transmembrane domains 5 and 6, TRPV2 S5‐S6) mediate cation influx, as TRPV2^E594/604Q^ (in which E594 and E604 were mutated into glutamine) completely lost the ion channel activity (Figure 3D).^[^ ^31^ ^]^ We next mapped the key amino acid residue responsible for the Ca^2+^ permeability of TRPV2. Sequence analysis identified additional nine negatively charged amino acid residues (E556, E564, D565, E572, E579, E580, E581, D590, and E609) within the outer mouth and the pore region of TRPV2 (Figure S6C, Supporting Information). Mutation each of the glutamic acid residues into glutamine did not affect the ion channel activity of TRPV2 (Figure 3D–F; and Figure S6D–F, Supporting Information). However, mutation of E572 but not other glutamic acid residues into glutamine (TRPV2^E572Q^) completely lost the ability to increase Ca^2+^ signal in HEK293 cells after stimulation of CBD in the presence of 1.8 mm extracellular calcium (Figure 3G,H; and Figure S6G, Supporting Information). These data thus indicate a pivotal role for E572 residue of TRPV2 in Ca^2+^ entry into the cytosol.

**Figure 4:**
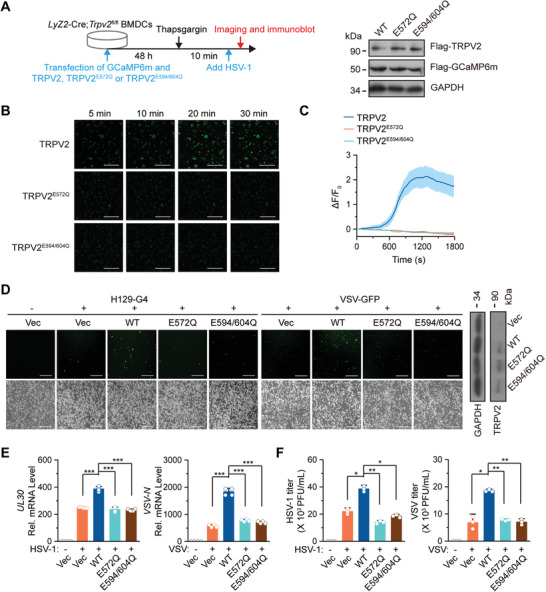
The Ca^2+^ permeability of TRPV2 is required for viral infection. A) A scheme of experiments (left) and immunoblot analysis (right) of GCaMP6m and TRPV2, TRPV2^E572Q^, or TRPV2^E594/604Q^ in *Lyz2*‐Cre;*Trpv2* ^fl/fl^ BMDCs for B,C). B) Fluorescent microscopy analysis of Ca^2+^ imaging in *Lyz2*‐Cre;*Trpv2* ^fl/fl^ BMDCs transfected with GCaMP6m and wild‐type TRPV2, TRPV2^E572Q^, or TRPV2^E594/604Q^ followed by infection of HSV‐1. C) Quantitative analysis of the GFP signals in *Lyz2*‐Cre;*Trpv2* ^fl/fl^ BMDCs treated as in B). D) Fluorescent microscopy imaging (left images) of *Lyz2*‐Cre;*Trpv2* ^fl/fl^ BMDCs that were transfected with an empty vector, wild‐type TRPV2, TRPV2^E572Q^, or TRPV2^E594/604Q^ followed by infection with H129‐G4 or VSV‐GFP for 12 h. The levels of TRPV2 or TRPV2 mutants in *Lyz2*‐Cre;*Trpv2* ^fl/fl^ BMDCs were analyzed by immunoblot assays (right panels). E) qRT‐PCR analysis of HSV‐1 *UL30* gene or VSV *N* gene in *Lyz2*‐Cre;*Trpv2* ^fl/fl^ BMDCs that were transfected with an empty vector, TRPV2, TRPV2^E572Q^, or TRPV2^E594/604Q^ and infected with HSV‐1 or VSV for 1 h followed by twice PBS wash and cultured in full medium for 12 h. F) Plaque assays analyzing HSV‐1 or VSV titers in the supernatants of *Lyz2*‐Cre;*Trpv2* ^fl/fl^ BMDCs treated as in E). *p < 0.05; **p < 0.01; ***p < 0.001 (two‐tailed student's *t*‐test in E,F). Graphs show mean ± S.D. in C, E,F). Scale bars represent 100 µm in B) and 200 µm in D). Data are representative of two B–F) independent experiments.

Because HSV‐1 infection increases cytosolic Ca^2+^ signal in a TRPV2‐dependent manner and E572 of TRPV2 mediates Ca^2+^ entry, we reasoned that TRPV2 mediates HSV‐1‐induced Ca^2+^ influx through E572. To test this hypothesis, we reconstituted wild‐type TRPV2, TRPV2^E572Q^, or TRPV2^E594/604Q^ into *Lyz2*‐Cre;*Trpv2* ^fl/fl^ BMDCs that were transfected with GCaMP6m. Forty‐eight hours later, the cells were treated with thapsigargin (1 µm) for 10 min to induce intracellular calcium release, and then infected with HSV‐1 followed by live cell imaging (**Figure** **4**A). Notably, reconstitution of TRPV2 but not TRPV2^E572Q^ or TRPV2^E594/604Q^ into *Lyz2*‐Cre;*Trpv2* ^fl/fl^ BMDCs increased the Ca^2+^ signal after HSV‐1 infection (Figure 4B,C). Collectively, these data indicate that E572 of TRPV2 mediates HSV‐1‐triggered Ca^2+^ influx in myeloid cells.

### 2.7 The Ca^2+^ Permeability of TRPV2 Mediates Viral Infection

We next examined whether the calcium permeability of TRPV2 affected viral infection in myeloid cells. The *Lyz2*‐Cre;*Trpv2* ^fl/fl^ BMDCs were reconstituted with an empty vector, wild‐type TRPV2, TRPV2^E572Q^, or TRPV2^E594/604Q^ and infected with wild‐type or H129‐G4) or VSV (VSV‐GFP) followed by fluorescent microscopy imaging, qRT‐PCR or plaque assays. The results suggested that reconstitution of wild‐type TRPV2 but not TRPV2^E572Q^ or TRPV2^E594/604Q^ into *Lyz2*‐Cre;*Trpv2* ^fl/fl^ BMDCs rescued HSV‐1 and VSV infection as monitored by the GFP signals in the cells, the expression levels of HSV‐1 *UL30* gene and VSV *N* gene in cells, and the viral titers in the supernatants (Figure 4D–F). In addition, the viral infection was comparable between *Lyz2*‐Cre;*Trpv2* ^fl/fl^ BMDCs reconstituted with TRPV2^E572Q^ and those with TRPV2^E594/604Q^ (Figure 4D–F), indicating that the Ca^2+^ permeability of TRPV2 is essential for viral infection in myeloid cells.

TRPV1 is a member of TRPV subfamily ion channel that also exhibits high ability of calcium permeability but barely expressed in BMDCs (Figure S1A,B and Table S1, Supporting Information), and the D646, E648, and E651 of TRPV1 mediate its calcium permeability.^[^ ^32^ ^]^ Interestingly, we found that reconstitution of wild‐type TRPV1 but not TRPV1^D646N/E648/651Q^ into *Lyz2*‐Cre;*Trpv2* ^fl/fl^ BMDCs rescued the Ca^2+^ influx after HSV‐1 infection (Figure S7A–C, Supporting Information). Not surprisingly, reconstitution of wild‐type TRPV1 but not TRPV1^D646N/E648/651Q^ into *Lyz2*‐Cre;*Trpv2* ^fl/fl^ BMDCs restored H129‐G4, HSV‐1, or VSV infections (Figure S7D–F, Supporting Information). Taken together, these data demonstrate that TRPV channels‐mediated Ca^2+^ influx facilitates viral infection in myeloid cells.

### 2.8 Knockout of TRPV2 Inhibits Virus Penetration

**Figure 5:**
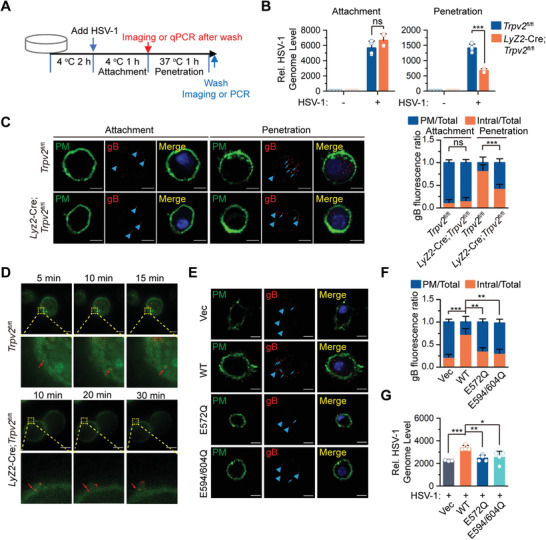
TRPV2 facilitates virus penetration dependently on its Ca^2+^ permeability. A) A scheme of experiments analyzing the attachment and the penetration of viruses. B) qPCR analysis of HSV‐1 genome of the attached and the penetrated HSV‐1 in *Trpv2* ^fl/fl^ and *Lyz2*‐Cre;*Trpv2* ^fl/fl^ BMDCs treated as in A). C) Fluorescent microscopy imaging (left) and quantification analysis (right) of the fluorescent signals of *Trpv2* ^fl/fl^ and *Lyz2*‐Cre;*Trpv2* ^fl/fl^ BMDCs treated as in A) followed by immunofluorescent staining with anti‐HSV‐1 gB protein (red) and CellMask Green (green). The arrowheads indicated gB signals on plasma membrane and the arrows indicated gB signals in the cytosol. D) Representative images captured from the a movie recording *Trpv2* ^fl/fl^ and *Lyz2*‐Cre;*Trpv2* ^fl/fl^ BMDCs that were infected with near‐infrared quantum dots‐encapsulated SV40 viruses. The arrows indicated SV40 viral particles. E,F) Fluorescent microscopy imaging E) and quantification analysis F) of the fluorescent signals of *Lyz2*‐Cre;*Trpv2* ^fl/fl^ BMDCs transfected with an empty vector, TRPV2, TRPV2^E572Q^, or TRPV2^E594/604Q^ followed by treatment as in C). The arrowheads indicated gB signals on plasma membrane and the arrows indicated gB signals in the cytosol. G) qPCR analysis of HSV‐1 genome in *Lyz2*‐Cre;*Trpv2* ^fl/fl^ BMDCs transfected with an empty vector, TRPV2, TRPV2^E572Q^, or TRPV2^E594/604Q^ followed by treatment as in A). *p < 0.05; **p < 0.01; ***p < 0.001; ns, not significant (two‐tailed student's *t*‐test in B,C, F,G). Graphs show mean ± S.D. in B,C, F,G). Scale bars represent 5 µm in C–E). PM, gB signals on plasma membrane; Intra, intracellular gB signals. Data are representative of three B–G) independent experiments.

Viral infection is a multistep process, including the early entry and replication stage and the late assembly and release stage.^[^ ^33^ ^]^ Interestingly, we found that the immediate early gene *UL54* was expressed in *Trpv2* ^fl/fl^ BMDCs and significantly downregulated in *Lyz2*‐Cre;*Trpv2* ^fl/fl^ BMDCs at the early stage of HSV‐1 infection (Figure S7G, Supporting Information). In contrast, the later transcription gene *UL52* of HSV‐1 was undetectable at the early stage of HSV‐1 infection (Figure S7G, Supporting Information). Both *UL54* and *UL52* were inhibited in *Lyz2*‐Cre;*Trpv2* ^fl/fl^ BMDCs compared to *Trpv2* ^fl/fl^ BMDCs at the late stage of HSV‐1 infection (Figure S7G, Supporting Information), indicating that the early stage of HSV‐1 infection is inhibited by TRPV2 deficiency in myeloid cells. We next examined how knockout of TRPV2 affected the early infection process. The *Lyz2*‐Cre;*Trpv2* ^fl/fl^ BMDCs and the *Trpv2* ^fl/fl^ BMDCs were precooled at 4 °C for 2 h and infected with HSV‐1 at 4 °C for 1 h to allow HSV‐1 to attach to the cells. The cells were washed with cold PBS and lysed to prepare HSV‐1 genomic DNA. Alternatively, the cells were incubated at 37 °C for 1 h to allow HSV‐1 penetration into cells. The cells were then washed with cold PBS followed by viral and host genomic DNA preparation, immunofluorescent staining or imaging analysis (**Figure** **5**A). Results from qPCR analysis suggested that the genomic DNA of attached HSV‐1 was comparable between the *Lyz2*‐Cre;*Trpv2* ^fl/fl^ BMDCs and the *Trpv2* ^fl/fl^ BMDCs, whereas the genomic DNA of penetrated HSV‐1 was significantly less in *Lyz2*‐Cre;*Trpv2* ^fl/fl^ BMDCs than in *Trpv2* ^fl/fl^ BMDCs (Figure 5B). Further analysis with immunofluorescent staining and microscopy imaging assays confirmed that the attachment of HSV‐1 to *Lyz2*‐Cre;*Trpv2* ^fl/fl^ BMDCs was comparable to *Trpv2* ^fl/fl^ BMDCs, whereas the cytosolic HSV‐1 signals were significantly decreased in *Lyz2*‐Cre;*Trpv2* ^fl/fl^ BMDCs compared to *Trpv2* ^fl/fl^ BMDCs (Figure 5C; and Figure S8A, Supporting Information). In addition, the penetration of near‐infrared quantum dots‐encapsulated SV40 viral particles into *Trpv2* ^fl/fl^ BMDCs was detected as early as 10–15 min after infection, whereas the penetration of SV40 viruses was substantially hampered in *Lyz2*‐Cre;*Trpv2* ^fl/fl^ BMDCs at 30 min after infection as indicated by live cell imaging with the high sensitivity structured illumination microscope (HiS‐SIM) (Figure 5D; and Videos S1 and S2, Supporting Information). These data together suggest that knockout of TRPV2 inhibited the penetration of viruses into but not the attachment of viruses to BMDCs.

We next examined whether TRPV2 played similar roles for RNA virus infection including sendai virus (SeV), VSV, or influenza A virus (IAV). The *Lyz2*‐Cre;*Trpv2* ^fl/fl^ BMDCs and the *Trpv2* ^fl/fl^ BMDCs were precooled at 4 °C for 2 h and infected with SeV, VSV, or IAV at 4 °C for 1 h to allow the viruses to attach to the cells. The cells were washed with cold PBS and lysed to prepare viral genomic and host RNA. Alternatively, the cells were incubated at 37 °C for 1 h to allow viruses penetration into cells. The cells were then washed with cold PBS followed by viral genomic RNA preparation. Results from qRT‐PCR analysis suggested that the levels of VSV *N* gene, SeV *NP* gene, or IAV *NP* gene of the attached viruses were comparable between the *Lyz2*‐Cre;*Trpv2* ^fl/fl^ BMDCs and the *Trpv2* ^fl/fl^ BMDCs, whereas the levels of VSV *N* gene, SeV *NP* gene, or IAV *NP* gene of the penetrated viruses were significantly lower in *Lyz2*‐Cre;*Trpv2* ^fl/fl^ BMDCs than in *Trpv2* ^fl/fl^ BMDCs (Figure S8B, Supporting Information). Similarly, the levels of VSV *N* gene of the attached VSV were comparable between the *Lyz2*‐Cre;*Trpv2* ^fl/fl^ peritoneal macrophages or Flt3L‐genrated cDCs and the *Trpv2* ^fl/fl^ counterparts, whereas the levels of VSV *N* gene of the penetrated VSV were significantly lower in *Lyz2*‐Cre;*Trpv2* ^fl/fl^ peritoneal macrophages and Flt3L‐genrated cDCs than in *Trpv2* ^fl/fl^ cells (Figure S8C, Supporting Information). These data together suggest that TRPV2 mediates the penetration of a broad spectrum of viruses into myeloid cells.

### 2.9 TRPV2 Facilitates Virus Penetration Dependently on Its Ca^2+^ Permeability

We next examined whether TRPV2 promoted virus penetration in a manner dependent on the Ca^2+^ permeability. The *Lyz2*‐Cre;*Trpv2* ^fl/fl^ BMDCs were reconstituted with an empty vector, wild‐type TRPV2, TRPV2^E572Q^, or TRPV2^E594/604Q^ followed by HSV‐1 attachment and penetration assays. The results from fluorescent staining and microscopy imaging assays suggested that the cytosolic HSV‐1 gB signal was significantly weaker and the percentages was lower in *Lyz2*‐Cre;*Trpv2* ^fl/fl^ BMDCs reconstituted with TRPV2^E572Q^ or TRPV2^E594/604Q^ than in those reconstituted with wild‐type TRPV2 (Figure 5E,F). Consistently, the genome of penetrated HSV‐1 was significantly lower in *Lyz2*‐Cre;*Trpv2* ^fl/fl^ BMDCs reconstituted with TRPV2^E572Q^ or TRPV2^E594/604Q^ than in those reconstituted with wild‐type TRPV2 (Figure 5G). In addition, the penetration of near‐infrared quantum dots‐encapsulated in the SV40 viral particles and the genomic RNA levels of the penetrated VSV were significantly inhibited in *Lyz2*‐Cre;*Trpv2* ^fl/fl^ BMDCs reconstituted with TRPV2^E572Q^ than in those reconstituted with wild‐type TRPV2 (Figure S8D,E and Videos S3 and S4, Supporting Information). These data suggest that TRPV2 mediates virus penetration dependently on its Ca^2+^ permeability.

To determine whether the calcium permeability was sufficient to restore virus penetration, we reconstituted wild‐type TRPV1 or TRPV1^D646N/E648/651Q^ into *Lyz2*‐Cre;*Trpv2* ^fl/fl^ BMDCs followed by infection with VSV or quantum dots‐encapsulated SV40 viruses and analysis with qRT‐PCR or HiS‐SIM live cell imaging. The results suggested that reconstitution of TRPV1 but not TRPV1^D646N/E648/651Q^ restored VSV and SV40 viruses penetration into *Lyz2*‐Cre;*Trpv2* ^fl/fl^ BMDCs (Figure S8D,E and Videos S5 and S6, Supporting Information). Collectively, these data together suggest that TRPV channel‐mediated Ca^2+^ permeability facilitates the penetration of viruses in myeloid cells.

### 2.10 TRPV2 Facilitates the Mobility of Cell Membrane Dependently on Its Ca^2+^ Permeability

Although virus‐triggered Ca^2+^ influx induces rapid rearrangement of cytoskeleton that promotes the dynamics of cell membrane for the penetration of viruses,^[^ ^34^, ^35^ ^]^ infections of VSV, IAV, SeV, or zika virus (ZIKV) did not trigger Ca^2+^ influx in *Trpv2* ^fl/fl^ BMDCs (Figure S9A,B, Supporting Information). The penetration of VSV, IAV, or SeV was substantially inhibited in various *Lyz2*‐Cre;*Trpv2* ^fl/fl^ myeloid cells compared to the *Trpv2* ^fl/fl^ counterparts (Figure S8B,C, Supporting Information). In addition, reconstitution of wild‐type TRPV2 or TRPV1 but not TRPV2^E572Q^ or TRPV1^D646N/E648/651Q^ into *Lyz2*‐Cre;*Trpv2* ^fl/fl^ BMDCs restored the penetration of VSV (Figure S8E, Supporting Information). These data indicate that the Ca^2+^ permeability of TRPV channels in myeloid cells under homeostatic conditions facilitates viral infections. In support of this notion, we found that the homeostatic intracellular Ca^2+^ concentrations were significantly reduced in *Lyz2*‐Cre;*Trpv2* ^fl/fl^ BMDCs compared to *Trpv2* ^fl/fl^ counterparts, which was restored by reconstitution of TRPV2 or TRPV1 but not TRPV2^E572Q^ or TRPV1^D646N/E648/651Q^ (Figure S9C, Supporting Information).

**Figure 6:**
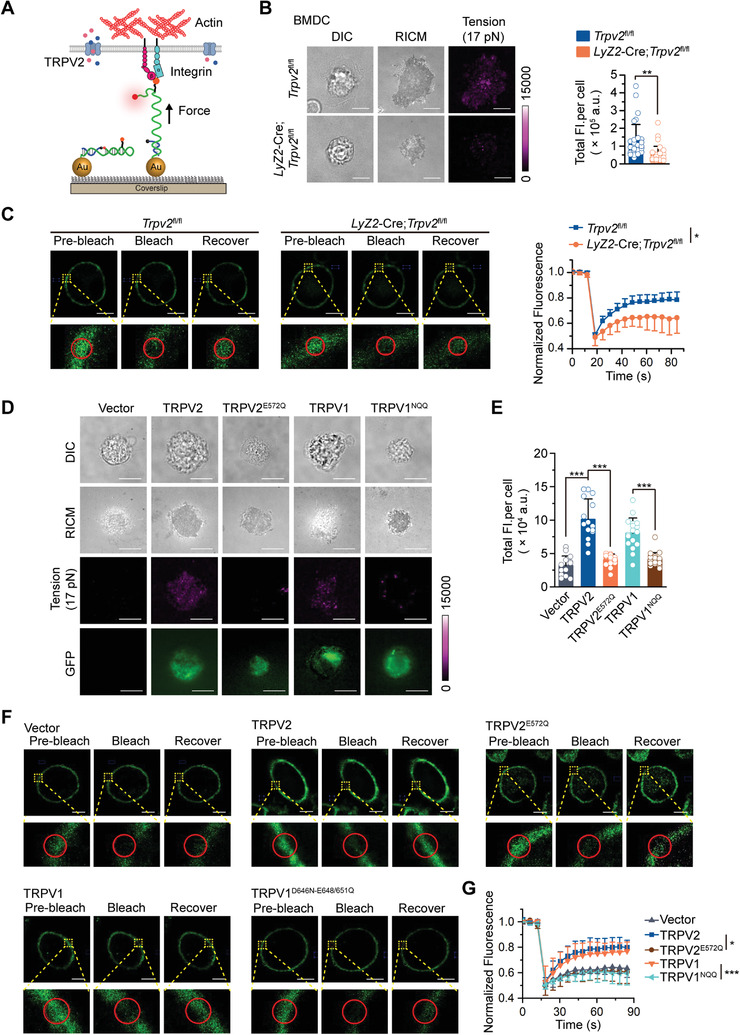
TRPV2 facilitates the tension and the mobility of cell membrane dependently on its Ca^2+^ permeability. A) Schematic illustrating the interaction between cells and the probe‐labeled surface. Detection of the fluorescence reflected the mechanic force of cell membrane exerted by the actin filaments and integrin. B) Representative images of differential interference contrast microscopy (DIC), reflection interference contrast microscopy (RICM, which can reflect the cell adherent area), and total internal reflection fluorescence (TIRF) microscopy (left) and statistic total fluorescent intensities (right) of *Trpv2* ^fl/fl^ and *Lyz2*‐Cre;*Trpv2* ^fl/fl^ BMDCs that were seeded on a 17 pN DNA probe surface. C) Representative images (left) and quantitative analysis (right) of FRAP in the cell membranes of *Trpv2* ^fl/fl^ and *Lyz2*‐Cre;*Trpv2* ^fl/fl^ BMDCs. D,E) Representative images of DIC, RICM, and TIRF microscopy D) and statistic total fluorescent intensities E) of *Lyz2*‐Cre *Trpv2* ^fl/fl^ reconstituted with Vector, TRPV2, TRPV2^E572Q^, TRPV1, and TRPV1^NQQ^ BMDCs that were seeded on a 17 pN DNA probe surface. F,G) Representative images F) and quantitative analysis G) of FRAP in the cell membranesof *Lyz2*‐Cre;*Trpv2* ^fl/fl^ BMDCs that were reconstituted with Vector, TRPV2, TRPV2^E572Q^, TRPV1, or TRPV1^D646N/E648/651Q^. *p < 0.05; **p < 0.01; ***p < 0.001 (two‐tailed student's *t*‐test in B,C,E,G). Graphs show mean ± S.D. in B,C,E,G). Scale bars represent 10 µm in B,D) and 5 µm in C,F). Data are representative of three B–F) independent experiments.

Viral infections trigger highly ordered movement of cell membrane that is controlled by the mechanic force by an actin filament bundle underneath the membrane.^[^ ^36^, ^37^, ^38^ ^]^ We next adopted a DNA‐based tension probe to measure the integrin force on the cell membrane of *Trpv2* ^fl/fl^ and *Lyz2*‐Cre;*Trpv2* ^fl/fl^ BMDCs that was exerted by the actin filament (Figure **6**A).^[^ ^39^ ^]^ The threshold of probe hairpin open and fluorescence recovery was >17 pN.^[^ ^39^ ^]^ The results suggested that the total florescent intensities of mechanical force of each *Lyz2*‐Cre;*Trpv2* ^fl/fl^ BMDC were significantly lower than those of each *Trpv2* ^fl/fl^ BMDC (Figure 6B). In addition, similar results were obtained with *Lyz2*‐Cre;*Trpv2* ^fl/fl^ and *Trpv2* ^fl/fl^ Flt3L‐cDCs, peritoneal macrophages and splenic DCs (Figure S9D–G, Supporting Information), indicating that TRPV2 promotes actin‐exerted tensile force on the cell membrane under homeostatic conditions. Importantly, results from transcriptomic analyses and flow cytometry assays suggested that the expression levels of integrins and the surface *α* integrins CD11c and CD11b were comparable between *Lyz2*‐Cre;*Trpv2* ^fl/fl^ and *Trpv2* ^fl/fl^ BMDCs or BMDMs (Figure S2G and Table S1, Supporting Information). Collectively, these data suggest that knockout of TRPV2 in myeloid cells reduces the tensile force of cell membrane.

Cytochalasin D (CytoD) is a potent inhibitor of actin polymerization and treatment of CytoD eliminates the tension signals of the probes.^[^ ^39^ ^]^ Interestingly, we found that treatment of CytoD significantly inhibited the fluorescence recovery in cell membrane of *Trpv2* ^fl/fl^ BMDCs in our fluorescence recovery after photobleaching (FRAP) and live cell imaging assays (Figure S9H, Supporting Information). In contrast, treatment of nocodazole (Noco) that inhibits the polymerization of microtubules did not affect the fluorescence recovery in cell membrane of *Trpv2* ^fl/fl^ BMDCs after photobleaching (Figure S9H, Supporting Information), indicating that the actin filament but not the tubulin controls the tensile force and the mobility of cell membrane. Consist with this notion, we found that the fluorescence recovery of the bleached fluorescence of the cell membrane was substantially inhibited in *Lyz2*‐Cre;*Trpv2* ^fl/fl^ BMDCs and BMDMs compared to the *Trpv2* ^fl/fl^ counterparts (Figure 6C; and Figure S9I, Supporting Information). These data together indicate that TRPV2 positively regulates the tension and mobility of cell membrane of myeloid cells.

We next examined whether TRPV2 regulated the mobility of cell membrane dependently on its Ca^2+^ permeability. The *Lyz2*‐Cre;*Trpv2* ^fl/fl^ BMDCs were reconstituted with wild‐type TRPV2, TRPV2^E572Q^, TRPV2^E594/604Q^, wild‐type TRPV1, or TRPV1^D646N/E648/651Q^ followed by the measurement of the tensile force by which the cell membrane attached to the probe‐labeled glass or by FRAP analysis. The results showed that reconstitution of GFP‐tagged TRPV2 or TRPV1 restored the tensions, whereas reconstitution of GFP‐tagged TRPV2^E572Q^, TRPV2^E594/604Q^, or TRPV1^D646N/E648/651Q^ failed to do so (Figure 6D,E). Consistently, the recovery of bleached fluorescence of the cell membrane was substantially restored in *Lyz2*‐Cre;*Trpv2* ^fl/fl^ BMDCs reconstituted with TRPV2 or TRPV1 but not with TRPV2^E572Q^ or TRPV1^D646N/E648/651Q^ (Figure 6F,G). Taken together, these data demonstrate that TRPV2 in myeloid cells positively regulates the tensile force and the mobility of cell membrane in a manner dependent on its Ca^2+^ permeability under homeostatic conditions.

### 2.11 LRMDA Functions Downstream of TRPV2 for the Mobility of Cell Membrane

**Figure 7:**
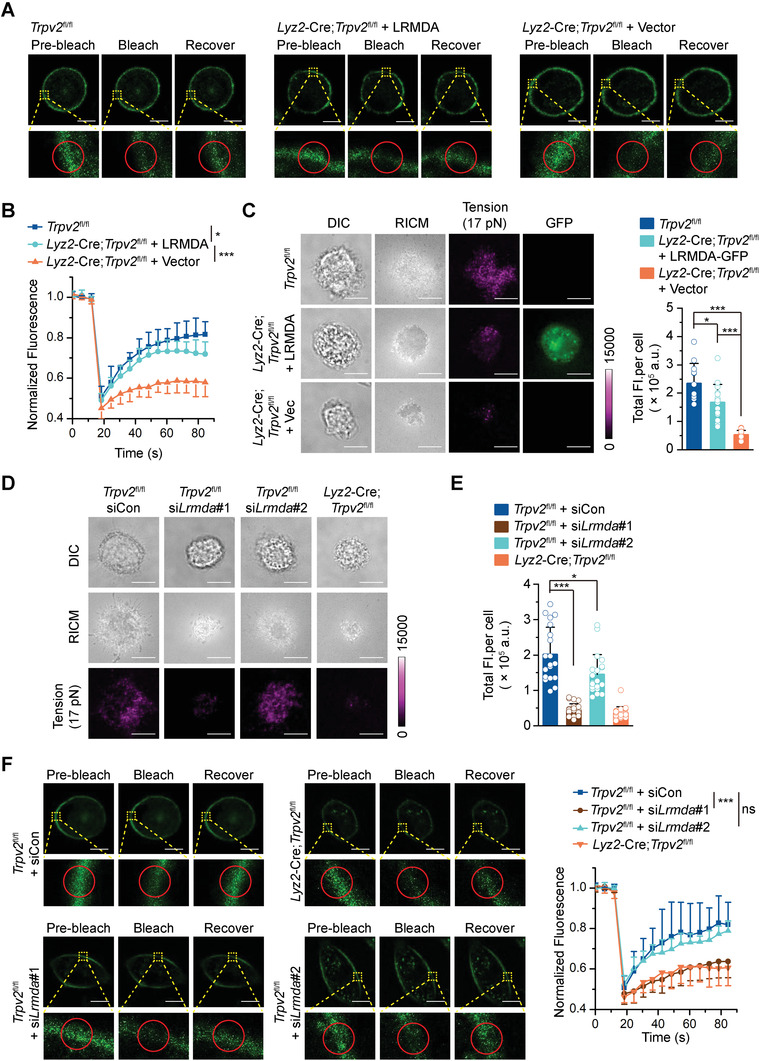
LRMDA functions downstream of TRPV2 for the mobility of cell membrane. A,B) Representative images A) and quantitative analysis B) of FRAP in the cell membranes of *Trpv2* ^fl/fl^ BMDCs and *Lyz2*‐Cre;*Trpv2* ^fl/fl^ BMDCs that were reconstituted with Vector or LRMDA. C) Representative images of DIC, RICM, and TIRF microscopy (left) and statistic total fluorescent intensities (right) of *Trpv2* ^fl/fl^ BMDCs and *Lyz2*‐Cre *Trpv2* ^fl/fl^ BMDCs reconstituted with LRMDA‐GFP or vector, associated tension signals reported by 17 pN tension probe. D,E) Representative images of DIC, RICM, and TIRF microscopy D) and statistic total fluorescent intensities E) of *Lyz2*‐Cre *Trpv2* ^fl/fl^ BMDCs and *Trpv2* ^fl/fl^ BMDCs transfected with siCon, si*Lrmda*#1, or si*Lrmda*#2. F) Representative images (left) and quantitative analysis (right) of FRAP in the cell membranes of *Lyz2*‐Cre *Trpv2* ^fl/fl^ BMDCs and *Trpv2* ^fl/fl^ BMDCs transfected with siCon, si*Lrmda*#1, or si*Lrmda*#2. *p < 0.05; **p < 0.01; ***p < 0.001; ns, not significant (two‐tailed student's *t*‐test in B,C, E,F). Graphs show mean ± S.D. in B,C, E,F). Scale bars represent 5 µm in A,F) and 10 µm in C,D). Data are representative of three A–F) independent experiments.

To figure out how TRPV2 deficiency impaired the tension and the mobility of cell membrane, we performed transcriptome mRNA sequencing assays with *Lyz2*‐Cre;*Trpv2* ^fl/fl^ and *Trpv2* ^fl/fl^ BMDCs and BMDMs. Analysis of the transcriptome data identified 4 genes significantly downregulated in *Lyz2*‐Cre;*Trpv2* ^fl/fl^ BMDCs and BMDMs compared to the *Trpv2* ^fl/fl^ counterparts (*Lyz2*, *Trpv2*, *Lrmda*, and *Gm15446*) (Figure S10A and Table S1, Supporting Information), which was confirmed by qRT‐PCR analysis (Figure S10B, Supporting Information). In addition, reconstitution of TRPV2 but not TRPV2^E572Q^ restored *Lrmda* expression in *Lyz2*‐Cre;*Trpv2* ^fl/fl^ BMDCs (Figure S10C, Supporting Information), indicating that the Ca^2+^ permeability of TRPV2 is required for the expression of *Lrmda* in myeloid cells under homeostatic conditions. Results from total internal reflection fluorescence (TIRF) microscopy assays suggested that a portion of LRMDA was closely located to the cell membrane in BMDCs and BMDMs (Figure S10D, Supporting Information). In addition, our semiquantitative liquid chromatography‐mass spectrometry assays and bioinformatics analyses suggested that the potential LRMDA‐interacting proteins were enriched in regulation of actin cytoskeleton, actin filament organization and viral infection pathways (Figure S10D–F and Table S2, Supporting Information), indicating a potential role of cytoskeleton remodeling of LRMDA. Importantly, reconstitution of LRMDA in *Lyz2*‐Cre;*Trpv2* ^fl/fl^ BMDCs partially but significantly rescued the fluorescence recovery in cell membrane in FRAP assays and the tensile force of cell membrane as measured by the DNA‐tension probe (**Figure** **7**A–C). Conversely, knockdown of *Lrmda* in *Trpv2* ^fl/fl^ BMDCs significantly reduced the tensile force of cell membrane and inhibited the fluorescence recovery in cell membrane, and the inhibitory extent corresponded well to the knockdown efficiency of the two siRNAs (Figure 7D–F; and Figure S10G, Supporting Information). These data together suggest that the TRPV2‐mediated expression of LRMDA critically facilitates the tension and mobility of cell membrane in myeloid cells.

### 2.12 LRMDA Functions Downstream of TRPV2 for Virus Penetration

**Figure 8:**
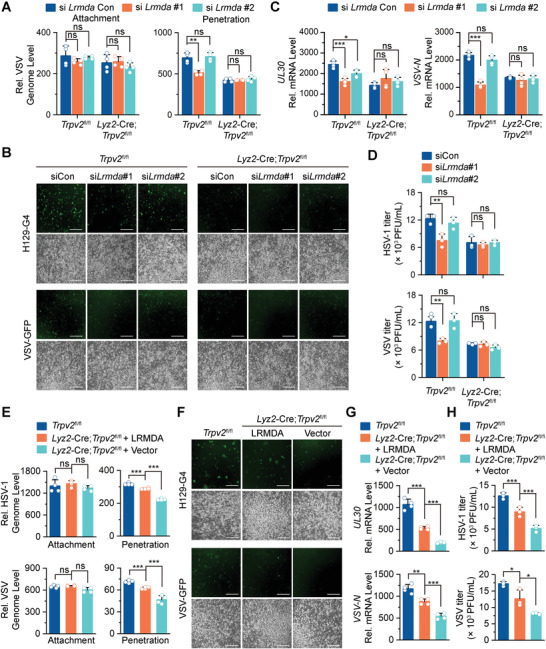
LRMDA facilitates viral infections downstream of TRPV2. A) qRT‐PCR analysis of genomic RNA of the attached VSV and the penetrated VSV in *Trpv2* ^fl/fl^ and *Lyz2*‐Cre *Trpv2* ^fl/fl^ BMDCs transfected with siCon, si*Lrmda*#1, or si*Lrmda*#2. B) Fluorescent microscopy imaging of GFP signals in *Trpv2* ^fl/fl^ and *Lyz2*‐Cre *Trpv2* ^fl/fl^ BMDCs that were transfected with siCon, si*Lrmda*#1, or si*Lrmda*#2 followed by infection with H129‐G4 or VSV‐GFP for 12 h. C) qRT‐PCR analysis of HSV‐1 *UL30* gene or VSV *N* gene in *Trpv2* ^fl/fl^ and *Lyz2*‐Cre *Trpv2* ^fl/fl^ BMDCs that were transfected with siCon, si*Lrmda*#1, or si*Lrmda*#2 followed by infection with HSV‐1 or VSV for 12 h. D) Plaque assays analyzing HSV‐1 or VSV titers in the supernatants of *Trpv2* ^fl/fl^ and *Lyz2*‐Cre *Trpv2* ^fl/fl^ BMDCs treated as in C). E) qPCR analysis of HSV‐1 genome (upper) or qRT‐PCR analysis of VSV genome (lower) of the attached and the penetrated HSV‐1 or VSV in *Trpv2* ^fl/fl^ BMDCs and *Lyz2*‐Cre;*Trpv2* ^fl/fl^ BMDCs that were transfected with LRMDA or an empty vector. F) Fluorescent microscopy imaging of *Trpv2* ^fl/fl^ BMDCs and *Lyz2*‐Cre;*Trpv2* ^fl/fl^ BMDCs that were transfected with LRMDA or an empty vector followed by infection with H129‐G4 or VSV‐GFP for 12 h. G,H) qRT‐PCR analysis of HSV‐1 *UL30* gene, or VSV *N* gene in G) and plaque assays of HSV‐1 and VSV titers in the supernatants of H) *Trpv2* ^fl/fl^ BMDCs and *Lyz2*‐Cre;*Trpv2* ^fl/fl^ BMDCs that were transfected with LRMDA or an empty vector followed by infection with HSV‐1 or VSV for 12 h. *p < 0.05; **p < 0.01; ***p < 0.001; ns, not significant (two‐tailed student's *t*‐test in A,C–E,G). Graphs show mean ± S.D. in A,C–E,G). Scale bars represent 200 µm in B,F). Data are representative of three A–G) independent experiments.

Because LRMDA promoted the tension and the mobility of cell membrane, we next examined whether LRMDA was involved in virus penetration downstream of TRPV2. As shown in **Figure** **8**A, knockdown of *Lrmda* significantly reduced the penetration of VSV in *Trpv2* ^fl/fl^ BMDCs but not in *Lyz2*‐Cre;*Trpv2* ^fl/fl^ BMDCs as monitored by qRT‐PCR analysis of VSV RNA. Consistently, viral infections were significantly compromised by knockdown of *Lrmda* in *Trpv2* ^fl/fl^ BMDCs but not in the *Lyz2*‐Cre;*Trpv2* ^fl/fl^ BMDCs as revealed by fluorescent microscopy and imaging, by plaque assays and by qRT‐PCR analyses (Figure 8B–D), indicating essential roles of LRMDA for HSV‐1 and VSV infections in myeloid cells. Consistently, reconstitution of LRMDA into *Lyz2*‐Cre;*Trpv2* ^fl/fl^ BMDCs significantly restored HSV‐1 and VSV penetration (Figure 8E). In addition, viral infections were substantially potentiated in *Lyz2*‐Cre;*Trpv2* ^fl/fl^ BMDCs reconstituted with LRMDA compared to those reconstituted with empty vector as determined by fluorescent microscopy and imaging, by plaque assays and by qRT‐PCR analyses (Figure 8F–G). Taken together, these data suggest that LRMDA functions downstream of TRPV2 to promote viral infections.

## 3 Discussion

Viral infections are one of the most common threats to human health. The entry of viruses is the first step for infection and depends on the dynamics of cell membrane.^[^ ^40^ ^]^ In this study, we have characterized previously unknown roles of TRPV2 in facilitating the penetration of viruses and maintaining the tensile force of cell membrane in myeloid cells. *Trpv2* was the most abundantly expressed *Trpv* gene in BMDCs and BMDMs. Though TRPV2 in BMDCs and BMDMs responded effectively to 2‐APB stimulation that was inhibited by SKF96365, it was dispensable for the differentiation and the homeostasis of immune cells in vitro and in mice. Interestingly, we found that knockout of TRPV2 significantly inhibited the penetration but not the attachment of multiple types of viruses, including HSV‐1, VSV, IAV, and SeV. Consistently, we further observed that the infection and replication of HSV‐1 and VSV was severely compromised in *Lyz2*‐Cre;*Trpv2* ^fl/fl^ myeloid cells and mice compared to the *Trpv2* ^fl/fl^ counterparts and that the *Lyz2*‐Cre;*Trpv2* ^fl/fl^ mice were more resistant to lethal HSV‐1 and VSV infections than the *Trpv2* ^fl/fl^ mice. These data suggest an indispensable role of myeloid TRPV2 in facilitating viral entry and virus‐caused lethality.

TRPV2 is a nonselective cation channel that induces Ca^2+^ influx into cells upon various stimulations or under homeostatic conditions. We further found that HSV‐1‐induced Ca^2+^ influx into *Lyz2*‐Cre;*Trpv2* ^fl/fl^ BMDCs and BMDMs was almost completely diminished compared to the *Trpv2* ^fl/fl^ counterparts, indicating that TRPV2 is activated by HSV‐1 infection. In addition, we characterized that the E572 of TRPV2 was a key site for Ca^2+^ permeability because TRPV2^E572Q^ lost the ability for Ca^2+^ influx in HEK293 cells after CBD treatment. Reconstitution of TRPV2^E572Q^ into *Lyz2*‐Cre;*Trpv2* ^fl/fl^ BMDCs failed to mediate Ca^2+^ influx after HSV‐1 infection, indicating that the E572 of TRPV2 mediates Ca^2+^ influx in myeloid cells in response to HSV‐1 infection. It has been demonstrated that virus‐triggered Ca^2+^ influx plays essential roles for viral entry and replication.^[^ ^14^, ^41^, ^42^, ^43^, ^44^ ^]^ A number of FDA‐approved drugs have been screened to target intracellular Ca^2+^ or Ca^2+^ channels for inhibiting viral infections.^[^ ^10^, ^45^, ^46^, ^47^ ^]^ Consistent with this notion, we observed that pharmacological inhibition of TRPV2 by SKF96365 in *Trpv2* ^fl/fl^ BMDCs and BMDMs or reconstitution of TRPV2^E572Q^ into *Lyz2*‐Cre;*Trpv2* ^fl/fl^ BMDCs inhibited the penetration of HSV‐1. TRPV1 is another TRPV channel that mediates Ca^2+^ influx but is barely expressed in BMDCs and BMDMs. Interestingly, we found that reconstitution of wild‐type TRPV1 but not TRPV1^D646N/E648/651Q^ (which lost the Ca^2+^ permeability) into *Lyz2*‐Cre;*Trpv2* ^fl/fl^ BMDCs almost fully restored HSV‐1‐induced Ca^2+^ influx and the penetration and infection of HSV‐1, indicating an indispensable role of TRPV channels‐mediated Ca^2+^ influx for the entry of HSV‐1. A recent study has shown that HSV‐1 gD interacts with TRPC1 and HSV‐1 infection induces Ca^2+^ influx though TRPC1 in HEp‐2 and MEF cells.^[^ ^48^ ^]^ Currently, we have little clue about how myeloid TRPV2 (or the reconstituted TRPV1) was activated by HSV‐1 infection, which might involve similar mechanisms as HSV‐1 does for TRPC1 and requires a thorough investigation with the cell‐type specific conditional knockout mice. Nonetheless, our findings suggest that TRPV2‐mediated Ca^2+^ influx facilitates the entry of HSV‐1 in myeloid cells.

Interestingly, however, we observed that infection of VSV, IAV, ZIKV, or SeV did not lead to robust Ca^2+^ influx in either *Trpv2* ^fl/fl^ or *Lyz2*‐Cre;*Trpv2* ^fl/fl^ BMDCs, indicating that TRPV2 is not activated by these viruses. However, the penetration of VSV, IAV, or SeV was substantially inhibited in *Lyz2*‐Cre;*Trpv2* ^fl/fl^ myeloid cells compared to the *Trpv2* ^fl/fl^ counterparts. We observed that the genomic RNA levels of the penetrated VSV were markedly lower in *Lyz2*‐Cre;*Trpv2* ^fl/fl^ BMDCs reconstituted with the empty vector, TRPV2^E572Q^ or TRPV2^D559Q/E604Q^ than in those with wild‐type TRPV2, but were comparable among the three groups of *Lyz2*‐Cre;*Trpv2* ^fl/fl^ BMDCs reconstituted with the empty vector, TRPV2^E572Q^ and TRPV2^E594/604Q^, indicating that TRPV2 facilitates the entry of viruses primarily dependent on its Ca^2+^ permeability. In addition, reconstitution of TRPV1 but not TRPV1^D646N/E648/651Q^ into *Lyz2*‐Cre;*Trpv2* ^fl/fl^ BMDCs restored the penetration of VSV. These findings point toward the idea that myeloid TRPV2‐mediated homeostatic Ca^2+^ facilitates the entry and infection of viruses and that selectively targeting the Ca^2+^ permeability of TRPV2 might represent potential therapeutic strategies for treatment of viruses‐caused diseases. In this context, the Ca^2+^ concentrations were substantially lower in *Lyz2*‐Cre;*Trpv2* ^fl/fl^ BMDCs than *Trpv2* ^fl/fl^ BMDCs under homeostatic conditions, which were restored by TRPV2 or TRPV1 but not TRPV2^E572Q^ or TRPV1^D646N/E648/651Q^.

Viruses hijack the cell membrane machinery for their entry.^[^ ^49^, ^50^, ^51^ ^]^ Interestingly, we found that the actin filament‐exerted tensile force of cell membrane that regulates the mobility of cell membrane was significantly reduced in various *Lyz2*‐Cre;*Trpv2* ^fl/fl^ myeloid cells compared to the *Trpv2* ^fl/fl^ counterparts. Such a process is dependent on the homeostatic Ca^2+^ influx mediated by TRPV channels, as reconstitution of TRPV1 or TRPV2 but not TRPV2^E572Q^ or TRPV1^D646N/E648/651Q^ into *Lyz2*‐Cre;*Trpv2* ^fl/fl^ BMDCs restored the membrane tension. In agreement with our findings, it has been shown that knockout of TRPV2 in macrophages impairs zymosan‐, immunoglobulin G‐, and complement‐mediated phagocytosis that is a process dependent on the dynamics of cell membrane.^[^ ^26^ ^]^

We further found that TRPV2‐mediated homeostatic Ca^2+^ permeability promoted the expression of *Lrmda* in BMDCs and BMDMs. Little function of LRMDA has been reported. However, a recent retrospective genome‐wide association study has revealed that human *LRMDA* gene has been implicated as a potential locus in non‐European patients susceptible to COVID‐19.^[^ ^52^ ^]^ In addition, TRPV2 has been reported to be associated with the spike protein of and mediate the entry of SARS‐CoV‐2 (severe acute respiratory syndrome coronavirus‐2) into macrophages.^[^ ^53^ ^]^ Our data showed that a portion of LRMDA was located closely to the cell membrane and potentially involved in the cytoskeleton remodeling pathways. In support of this notion, reconstitution of LRMDA into *Lyz2*‐Cre;*Trpv2* ^fl/fl^ BMDCs partially but significantly restored the membrane tension and mobility. In addition, reconstitution of LRMDA partially restored the penetration and replication of VSV and HSV‐1 in *Lyz2*‐Cre;*Trpv2* ^fl/fl^ BMDCs compared to *Trpv2* ^fl/fl^ BMDCs, which might be due to yet‐to‐identify TRPV2‐mediated Ca^2+^‐dependent LRMDA‐independent regulatory mechanisms. Conversely, knockdown of LRMDA in *Trpv2* ^fl/fl^ BMDCs but not *Lyz2*‐Cre;*Trpv2* ^fl/fl^ BMDCs substantially impaired the tension and mobility of cell membrane and the penetration of HSV‐1 and VSV. Though the exact mechanisms by which homeostatic Ca^2+^ by TRPV2 mediated the expression of LRMDA and by which LRMDA regulated the tension of cell membrane are currently unclear, these available data have clearly suggested that the TRPV2‐Ca^2+^‐LRMDA axis promotes viral infections and that targeting the TRPV2‐Ca^2+^‐LRMDA axis would provide potential therapeutic interventions for the treatment of virus‐caused diseases.

## 4 Experimental Section

### Mice

The *Trpv2* ^fl/+^ mice were generated by Beijing Biocytogen Co., Ltd through CRISPR/Cas9‐mediated genome editing. In brief, the vectors encoding Cas9 (44 758, Addgene) and guide RNAs (5′‐GGCTGCACGCGTCCCTGACTCGG‐3′; 5′‐GAGCTATTGCCTTATGCCCCTG G‐3′) were in vitro transcribed into mRNA and gRNA followed by injection into the fertilized eggs that were transplanted into pseudopregnant mice. The targeted genome of F0 mice was amplified with PCR and sequenced and the chimeras were crossed with WT C57BL/6 mice to obtain the F1 *Trpv2* ^fl/+^ mice. Southern blot analysis was conducted with the tail DNA from F1 mice to confirm correct recombination and to exclude random insertions of the targeting vector. The *Lyz2*‐Cre mice and the wild‐type C57BL/6 were purchased from GemPharmatech Co., Ltd, Nanjing, China. Subsequently, the *Trpv2* ^fl/+^ mice were crossed with the *Lyz2*‐Cre mice to obtain the *Lyz2*‐Cre;*Trpv2* ^fl/fl^ mice. The genotyping primers are listed in Table S3 (Supporting Information). All mice were housed in the specific pathogen‐free animal facility at Wuhan University with a 12 h dark/12 h light cycle and fed with standard food and water. All animal experiments were in accordance with protocols approved by the Institutional Animal Care and Use Committee of Wuhan University (Approval NO. 21020A) and adhered to the Chinese National Laboratory Animal‐Guideline for Ethical Review of Animal Welfare. The animals were euthanatized with CO_2_ followed by various studies.

### Constructs, Reagents, and Antibodies

The wild‐type mouse TRPV2 and mouse TRPV1 cDNAs were generously provided by Dr. Feng Qin (State University of New York at Buffalo, Buffalo). For the expression in mammalian cells, the cDNAs of TRPV1, TRPV2, LRMDA, GCaMP6m, LRMDA‐GFP, LRMDA‐RFP, TRPV2‐GFP, TRPV1‐GFP, TRPV2‐RFP, and TRPV1‐RFP were amplified and cloned into the phage‐6‐tag vector or the RVKM vector. The mutants encoding TRPV1^D646N/E648/651Q^, TRPV2^E556Q^, TRPV2^E564Q^, TRPV2^D565Q^ TRPV2^E572Q^, TRPV2^E579Q^, TRPV2^E580Q^, TRPV2^E581Q^, TRPV2^D590Q^, TRPV2^E594Q^, TRPV2^E604Q^, TRPV2^E609Q^, TRPV2^E594/604Q^, TRPV1^D646N/E648/651Q^‐GFP, TRPV2^E572Q^‐GFP, TRPV2^E594/604Q^‐GFP, TRPV1^D646N/E648/651Q^‐RFP, TRPV2^E572Q^‐RFP, and TRPV2^E594/604Q^‐RFP were generated using the overlap‐extension polymerase chain reaction method and verified by DNA sequencing. H129‐G4 and VSV‐GFP viruses were previously described and kindly provided by Dr. Min‐Hua Luo (Wuhan Institute of Virology, Chinese Academy of Sciences).^[^ ^54^ ^]^

HRP‐conjugated goat‐anti mouse or rabbit IgG (Thermo Scientific, PA1‐86717 and SA1‐9510), HRP‐conjugated mouse anti‐FLAG (Sigma, A8592), mouse anti‐FLAG (Sungene, KM8002), anti‐*β*‐Actin (Sungene, KM9001), anti‐GAPDH (Sungene, KM9002), anti‐Tubulin (Sungene, KM9003), Rabbit anti‐TRPV2 (Alomone labs, ACC‐032), anti‐HSV‐1/2 gB (SANTA, sc‐56987), SKF96365 (MCE, 130495‐35‐1), 2‐APB (Sigma, D9754), ionomycin (Aladdin, 56092‐81‐0), GM‐CSF (Peprotech, 315‐03), M‐SCF(Peprotech, 315‐02), Flt3L (PeproTech, 250‐31L), and CellMask Green plasma membrane stain (Invitrogen, C37608) were purchased from the indicated manufactures.

### Western Blot

The experiments were performed as previously described.^[^ ^55^, ^56^ ^]^ In brief, cells were lysed in Nonidet P‐40 lysis buffer containing 150 mm NaCl, 1 mm EDTA, 1% Nonidet P‐40, and 1% protease and phosphatase inhibitor cocktail (Biotool). Lysates were cleared of debris by centrifugation at 12 000 rpm for 10 min. The supernatants were collected, mixed with the 5x Laemmli Sample Buffer (0.3 m Tris‐HCl pH 6.8, 10% SDS, 50% glycerol, 25% beta‐mercaptoethanol, and 0.05% bromophenol blue), and boiled at 95 °C prior to SDS‐PAGE and immunoblot analysis was performed with the appropriate antibodies. The antibodies were diluted (≈1:500–1:2000) in ≈3–5% (wt/vol) fat‐free milk (BD Biosciences) or 1% BSA (Sigma) in TBS.

### Flow Cytometry Analysis

Single‐cell suspension was resuspended in FACS buffer (PBS, 1%BSA) and blocked with anti‐mouse CD16/32 antibodies for 10 min prior to staining with antibodies against surface markers. Staining antibodies against CD3, CD4, CD8, CD19, CD25, CD44, CD62L, CD11c, CD11b, B220, Ly6G, Ly6C, MHC‐II, and F4/80 were purchased from Biolegend or Sungene. For the preparation of spleen DCs, spleen was grinded by syringe plunger and the red blood cells were lysed with ACK lysis buffer. Debris was removed by passing cells through a 70 µm strainer and cells were counted via Countess II FL Automated Cell Counter. The cells were stained with CD11c, CD3, and CD19 for 30 min at 4 °C. Flow cytometry sorting was performed to obtain the CD3^−^CD19^−^CD11c^+^ splenic DCs. Viral infection was monitored by flow cytometry of the GFP signals. Flow cytometry data were acquired on Aria or LSRFortessaX20 flow cytometer (BD Biosciences) and analyzed with FlowJo X software (TreeStar).

### Viral Infection and Analysis

For qRT‐PCR analysis, cells were seeded into 12‐well plates (1−3 × 10^6^ cells per well) and infected with the specified viruses for the indicated time points. For viral infection assays, the cells (1−3 × 10^6^) were infected with VSV‐GFP, H129‐G4, VSV, or HSV‐1. One hour later, the supernatants were removed and the cells were washed twice with 1 mL prewarmed PBS followed by culture in full medium for 12 h. Viral infections were analyzed by flow cytometry and fluorescent microscopy imaging. The expression levels of HSV‐1 *UL30* gene and VSV *N* gene were determine with qRT‐PCR analysis. The titers of viruses in the supernatants were determined through plaque assays. For the infection of mice, age‐ and sex‐matched *Trpv2* ^fl/fl^ and *Lyz2*‐Cre;*Trpv2* ^fl/fl^ mice were intraperitoneally injected with VSV (1−2 × 10^7^ PFU per mouse) or HSV‐1 (2−5 × 10^6^ PFU per mouse) and the survival of animals was monitored every day. Alternatively, the hearts, spleens, kidneys, or brains were collected for qRT‐PCR analysis or plaque assays at 4 days after infection.

### Plaque Assay

The supernatants of BMDCs or BMDMs cultures and the homogenates (and the serial dilutions) of brains from infected mice were used to infect monolayers of Vero cells. One hour later, the supernatants or the homogenates were removed and the infected Vero cells were washed with prewarmed PBS twice followed by incubation with Dulbecco's modified Eagle's medium (DMEM) containing 1% methylcellulose for 48 h. The cells were fixed with 4% paraformaldehyde for 15 min and stained with 1% crystal violet for 30 min before counting the plaques.

### Detection of the Attached and the Penetrated Viruses

The attachment and the penetration of viruses were assayed as previously described.^[^ ^48^, ^57^ ^]^ For the detection of viral attachment, the cells were precooled at 4 °C for 2 h and then incubated with viruses at 4 °C for 1 h. Subsequently, the cells were washed to remove unbounded viruses and subject to preparation of DNA or RNA. For the detection of viral penetration, the PBS‐washed cells were immediately moved to incubators and cultured at 37 °C for 1 h. Subsequently, the cells were lysed to prepare viral and host DNA or RNA.

Preparation of HSV‐1 genomic DNA and host cell DNA was carried out by TIANamp Genomic DNA Kit (TIANamp, DP304‐03). HSV‐1 genome copy number was determined by qPCR with host *β‐Actin* genome as an internal control. The genomes of RNA viruses and host mRNAs were prepared by TRIzol (Takara, T9108). The first‐strand cDNA was reverse‐transcribed with All‐in‐One cDNA Synthesis SuperMix (Biotool). The viral genome copy number was examined by qRT‐PCR (Biotool). The primers for detection of genomes or genes of viruses and host cells were listed in Table S4 (Supporting Information).

The attachment and the penetration of HSV‐1 were additionally assayed by the following experiments. The cells (7 × 10^5^) were precooled at 4 °C for 2 h and then incubated with HSV‐1 (MOI  =  1) or EdU (5‐ethynyl‐2′‐deoxyuridine)‐labeled HSV‐1 (MOI  =  1) at 4 °C for 1 h followed by PBS wash for twice. The cells were either fixed with 4.0% formaldehyde (for immunofluorescent staining) for 15 min at room temperature or immediately moved to incubators and cultured at 37 °C for 12 h followed by fixation. The cells were subject to immunofluorescent staining and fluorescent microscopy imaging.

The near‐infrared quantum dots‐encapsulated SV40 viral particles were kindly provided by Dr. Zongqiang Cui (Wuhan Institute of Virology, CAS) as previous described and applied for the analysis of the entry of SV40 viruses.^[^ ^58^ ^]^ The cells were labeled with CellMask Green and cultured in a live imaging chamber with 5% CO_2_ at 37 °C under the LSM880 Zeiss fluorescent microscope (High Sensitivity Structured Illumination Microscope.). Immediately after CellMask Green labeling, the cells were infected with the SV40 virus and elicited for 30–60 min with the 488 nm laser. The cells were lively imaged by recording the emission signals at 493 and 589 nm.

### Transmission Electron Microscopy

The samples for ultrathin section were prepared as previously described.^[^ ^56^ ^]^ Briefly, the cells were fixed with 3.0% formaldehyde plus 1.5% glutaraldehyde in 100 mm phosphate buffer (PB) containing 2.5% sucrose (pH = 7.4) for 1 h at room temperature. After washing for three times with PB buffer containing 2.5% sucrose, the cells were lifted and pelleted at progressively increasing g forces (1000 g for 5 min, 3000 g for 5 min, 6000 g for 5 min, and finally 12 000 g for 5 min) to minimize the shear of cells. Then, the samples were postfixed with 2% osmium tetroxide (OsO_4_) for 1 h on ice, protected from light. The samples were rinsed once with uranyl acetate and incubated in uranyl acetate for overnight in the dark at room temperature. At the second day, samples were further rinsed in double‐distilled water followed by a quick rinse in cold (4 °C) 50% ethanol. Then, the samples were dehydrated with graded series of cold ethanol (70%, 95%, and 100%). After washed three times with epoxypropane, the samples were infiltrated sequentially in 1:1 v:v epoxypropane/epoxy resin for 4 h, 1:2 v:v epoxypropane/epoxy resin (overnight), 100% fresh epoxy resin for 4 h, and finally 100% fresh epoxy resin for 48 h at 60 °C for polymerization. The ultrathin sections on copper grids were poststained in Sato lead Sodium citrate for 1 min and observed using a JEM‐1400 plus electron microscope operated at 100 kV.

### Immunofluorescent Staining and Confocal Microscopy Analysis

The cells were fixed with 4% paraformaldehyde for 15 min, and permeabilized with 0.5% saponin in PBS for 5 min (optional). After three washed with PBS, the cells were blocked in 1% BSA, and incubated overnight at 4 °C with PBS containing 1% BSA and primary antibody (1:100). The cells were washed with PBS for three times followed by incubation with fluorescence‐labeled secondary antibody (1:300) for 1 h. Images were acquired with a Leica SP8 fluorescence microscope and quantified with the software ImageJ.

### EdU (5‐ethynyl‐2′‐deoxyuridine) Labeling and Staining

EdU‐labeled HSV‐1 were obtained by propagating HSV‐1 in Vero cells cultured in DMEM containing 10% FBS and 25 µm EdU (C00052, RIBOBIO). The cells and the supernatants were collected for three freeze‐thaw cycles to obtain EdU‐labeled HSV‐1. The BMDCs were incubated with EdU‐labeled HSV‐1 and cell Mask Green at 4 °C for 1 h (attachment) or at 37 °C for 15–30 min (penetration). Subsequently, the cells were then fixed in 4% paraformaldehyde and stained with Apollo reaction cocktail (C00031, RIBOBIO) at room temperature for 30 min. Images were captured on a Leica SP8 fluorescence microscope and processed with the ImageJ software for quantitative analysis.

### In Vitro Differentiation and Culture of BMDCs, BMDMs, and Flt3L‐cDCs

For BMDCs and BMDMs preparation, femurs and tibias were dissected from *Trpv2* ^fl/fl^ and *Lyz2*‐Cre;*Trpv2* ^fl/fl^ mice. Bone marrow was flushed from the bones, and the red blood cells were lysed with ACK lysis buffer. Debris was removed by passing cells through a 70 µm strainer and cells were counted via Countess II FL Automated Cell Counter. Approximately 5 × 10^6^ bone marrow cells were cultured in 100 mm dishes in DMEM (Thermo Fisher Scientific, MA) containing 10% heat‐inactivated fetal bovine serum (FBS, Gibco, Thermo Fisher Scientific) and 1% streptomycin‐penicillin at 37 °C in a humidified incubator gassed with 5% CO_2_. GM‐CSF (20 ng mL^−1^, Peprotech) and M‐CSF (10 ng mL^−1^, Peprotech) were added to the bone marrow cultures for differentiation of BMDCs and BMDMs, respectively. The medium was changed every 3 days. On day 7, cells were collected for subsequent analysis. For the evaluation of differentiation, the cells were stained with CD11c, CD11b, F4/80, MHC‐II followed by flow cytometry analysis. For the reconstitution of various plasmids, the cells were electroporated with the plasmids or infected with the peudopackaged lenti‐viruses or retroviruses followed by flow cytometry sorting. For the measurement of cell membrane tension assays, the BMDCs were stained with CD11c, CD11b, F4/48, and MHC‐II for 30 min at 4 °C. Flow cytometry sorting was performed to obtain the CD11c^+^CD11b^+^F4/80^−^MHC‐II^+^ cells.

For the preparation of cDCs, bone marrow cells were cultured in RPMI medium containing 10% FBS, 1% streptomycin‐penicillin, and recombinant mouse Flt3L (20 ng mL^−1^, PeproTech). The medium was changed every 3 days. On day 7, cells were stained with CD11c, CD11b, and B220 for 30 min at 4 °C Flow cytometry sorting was performed to obtain the CD11c^+^CD11b^+^B220^−^ cDCs. HEK293 cells were from the American Type Culture Collection, authenticated by STR locus analysis and tested for mycoplasma contamination.

### Isolation of Peritoneal Macrophages

Eight‐week old mice were intraperitoneally injected with ≈1–1.5 mL of sterile Brewer thioglycollate broth. Five days later, the mice were euthanized and the abdomen skin was carefully cut to expose the abdomen. PBS (5 mL twice) was injected into the peritoneal cavity and retrieved into a 15 mL tube followed by centrifuge for 5 min at 4 °C at 1500 r min^−1^ to pellet cells. The supernatant was discarded and the red blood cells were lysed with ACK lysis buffer. The resulted cells were washed with PBS, resuspended in 1 mL complete RPMI medium and counted on a hemacytometer. The resulted cells were cultured in complete RPMI medium in a humidified incubator with 5% CO_2_ at 37 °C for overnight followed by various experiments.

### Lentivirus‐Mediated Gene Transfer

HEK293 cells were transfected with the indicated plasmids along with the packaging vectors pSPAX2 and pMD2G. The medium was changed with fresh full DMEM medium (10% FBS, 1% streptomycin‐penicillin) at 8 h after transfection. Forty hours later, the supernatants were harvested to infect BMDCs and BMDMs followed by various analyses.

### qRT‐PCR and qPCR

Total RNA was extracted from cells using TRIzol (Takara, T9108), and the first‐strand cDNA was reverse‐transcribed with All‐in‐One cDNA Synthesis SuperMix (Biotool). Gene expression was examined with a Bio‐Rad CFX Connect system by a fast two‐step amplification program with 2x SYBR Green Fast qRT‐PCR Master Mix (Biotool). The value obtained for each gene was normalized to that of the gene encoding *β*‐Actin. The primers for the indicated genes or HSV‐1 genome were listed in Table S4 (Supporting Information).

### siRNA

The siRNAs targeting LRMDA was synthesized and transfected with Lipofectamine 2000 according to the manufacture's manual. Forty hours after transfection, the cells were harvested for various analyses. The siRNA sequences were listed as follows: control siRNA, 5′‐UUCUCCGAACGUGUCACGUTT‐3′; si*Lrmda*#1, 5′‐GUCCUGCAGAGAAAUUCCATT‐3′; si*Lrmda*#2, 5′‐GUGACAC CAUCCUUAGAGUTT‐3′. Specifically, FAM‐labeled siRNA and unlabeled siRNA (1:5) were simultaneously transfected into cells to determine that the examined cells were successfully transferred with siRNA.

### Fluorescence Recovery after Photobleaching (FRAP)

Live cells were stained with cell membrane mask Green (Invitrogen) at 37 °C for 10 min. Images were acquired with an LSM880 confocal microscope equipped with a live cell chamber (set at 37 °C and 5% CO_2_) and ZEN software (Zeiss) with a 63x oil immersion objective. The cells were excited with a 488 nm laser and the emission signals at 493 and 589 nm were recorded. Pictures were acquired with 16 bits image depth and 1024 × 1024 resolution, using a pixel dwell of ≈1.02 µs. Images were analyzed using ImageJ version 1.47 software.

### Live Cell Imaging

The inverted confocal microscope system (Zeiss LSM 880 and HiS‐SIM) was adopted for live imaging. For Zeiss LSM 880, the microscope was mounted on a Zeiss Axio Observer Z1 basic stand equipped with an incubator (XLmulti S1) to maintain the temperature at 37 °C and 5% CO_2_. The Plan‐Apochromat 20×/0.8 NA M27 objective (Zeiss) was used for imaging. Time‐lapse images were acquired every 10 s for 30 min. The laser excitation and filter were set for enhanced GFP using the settings detailed above. Image processing was performed with ZEN software (blue edition, Zeiss). HiS‐SIM (High Sensitivity Structured Illumination Microscope) is provided by the Guangzhou Computational Super‐resolution Biotech Co., Ltd.

### Glass Surface and DNA Probe Preparation

The procedure for glass surface functionalization and Au nanoparticles immobilization were slightly adapted from methods previously developed by the Salaita lab.^[^ ^39^ ^]^ Circular coverslips (25 mm diameter, 130 µm thickness) were rinsed and sonicated three times in nanopure water (18.2 MΩ cm), and further sonicated in acetone for 20 min. After drying in an oven, the coverslips were activated in the oxygen plasma for 10 min (30 s.c.c.m, 300 mttor) to achieve a hydroxylated surface. Subsequently, the coverslips were functionalized with amine groups by incubating the coverslips in ethanol with a 1% v/v (3‐Aminopropyl) triethoxysilane for 1 h. The amine‐modified coverslips were then rinsed in acetone and dried under a stream of N2. The slides were then annealed for 1 h at 80 °C. The coverslips were then passivated by covering with 200 µL of 0.1 m fresh sodium bicarbonate solution containing 5% w/v mPEG‐NHS (*M*_W_ = 2000) and 0.5% w/v lipoic acid‐PEG‐NHS (*M*_W_ = 3400). After overnight incubation at 4 °C, the surface was washed with nanopure water. Subsequently, the 14 nm 5 nm Au solution was added on the coverslip surface to incubate for 30 min at room temperature, followed by three rinses with nanopure water and. The nonspecific bound AuNPs were removed by sonication of the coverslips for 1 min in nanopure water. Finally, 200 nm DNA tension probe in PBS (10 mm sodium phosphate, 1 m NaCl, pH 7.2) was added onto the surface and incubated at room temperature for 1 h. DNA tension probe modified coverslips were rinsed by PBS solution to remove nonspecifically bound probes. These modified coverslips were then assembled into cell imaging chambers and immediately used for cell experiment.

### Electrophysiology

Conventional whole‐cell patch‐clamp recordings were performed using an Axopatch 200B amplifier (Molecular Devices, Sunnyvale, CA) through a BNC‐2090/MIO acquisition system (National Instruments, Austin, TX). Data acquisition was controlled by QStudio developed by Dr. Feng Qin at State University of New York at Buffalo. Currents were typically acquired at 5 kHz sampling frequency and low‐pass filtered at 1 kHz. Recording pipettes were pulled from borosilicate glass capillaries (World Precision Instruments, WPI), and fire‐polished to a resistance between 2 and 4 MΩ when filled with 150 mm NaCl solution. The compensation of pipette series resistance (> 80%) and capacitance was taken by using the built‐in circuitry of the amplifier, and the liquid junction potential between the pipette and bath solutions was zeroed prior to seal formation. All voltages were defined as membrane potentials with respect to extracellular solutions.

For whole‐cell recording, bath solution consisted of (mm): 140 NaCl, 5 KCl, 3 EGTA, and 10 HEPES, pH 7.4 adjusted with NaOH. The internal pipette solution contained the following (mm): 140 CsCl, 5 EGTA, 10 HEPES, pH 7.4 adjusted with CsOH. Channel activators were diluted into the recording solution at the desired final concentrations and applied to the cell of interest through a gravity‐driven local perfusion system. As determined by the conductance tests, the solution around a patch under study was fully controlled by the application of a solution with a flow rate of 100 µL min^−1^ or greater. All pharmacological experiments met this criterion. 2‐Aminoethyl diphenylborinate (2‐APB) was dissolved in DMSO to make a stock solution. The final concentration of DMSO did not exceed 0.3%, at which it did not affect the channel currents. Unless otherwise stated, all chemicals were obtained from Sigma (Millipore Sigma, St. Louis, MO). All patch‐clamp recordings were made at room temperature (22–24 °C).

### Ca^2+^ Imaging

Ca^2+^ imaging was performed as previously described with minor modifications.^[^ ^59^ ^]^ Cells were coexpressed with the GCaMP6m (a gift from Dr. Liangyi Chen, Peking Unversity, Beijing, China), and were washed twice with PBS. Fluorescent images were acquired under an inverted epifluorescence microscope (Olympus IX 73, Tokyo, Japan) equipped with a complete illumination system (Lambda XL, Sutter Instruments). Intracellular Ca^2+^ was measured using a cool CCD camera (CoolSNAP ES2, Teledyne Photometrics) which was controlled by Micro‐Manager 1.4 (Vale lab, UCSF) at 470 ± 22 nm excitation. The extracellular solution contained with 140 mm NaCl, 5 mm KCl, 1.8 mm CaCl_2_, and 10 mm HEPES, pH 7.4. Changes in intracellular Ca^2+^ levels were calculated by subtracting the basal fluorescence intensity (mean value collected for 10 s before agonist addition) from the fluorescence intensity after exposure to agonist. Fluorescence intensity in individual cells (region of interest) was recorded and analyzed by ImageJ.

In another set of experiments, cells were incubated with 2 µm Fluo‐3‐AM/PBS at 37 °C for 30 min, followed by PBS wash for three times at room temperature. The intensities of Fluo‐3 fluorescence were detected at an emission wavelength of 526 nm under an excitation wavelength of 488 nm with an Olympus fluorescent microscopy. First, a single cell was imaged by 10 consecutive pictures (with 2 s intervals) and the average fluorescence was recorded as basal fluorescence activated by cytoplasmic calcium (*F*). Then, the cell was imaged by 15 consecutive pictures (with 2 s intervals) under the perfusion with high calcium solution containing 1 µm ionomycin. The highest intensity of fluorescence was recorded as the maximum fluorescence (*F*_max_). Finally, the Ca^2+^ free 5 mm EGTA buffer was added and the cell was imaged by 15 pictures (with 2 s intervals). The average of lowest five fluorescence intensities was recorded as the minimum fluorescence (*F*_min_). [Ca^2+^]*i* was quantified in nm according to the following equation in which *K*_d_ is 400 nm at 37 °C: [Ca^2+^]*_i_* = *K*_d_·[*F* – *F*_min_]/[*F*_max_ – *F*].

### Liquid Chromatography‐Mass Spectrometry (LC‐MS) Analysis

The experiments were performed as previously described.^[^ ^60^ ^]^ HeLa cells were stably transfected with FLAG‐LRMDA or an empty vector were followed by coimmunoprecipitation assays with anti‐FLAG affinity gel (M2 beads). The immunoprecipitates were washed three times by 1 mL PBS and eluted by FLAG peptides. The elutions were subject to digestion with trypsin (Sigma, SLBF7596V) overnight at 37 °C. The resulting peptide mixtures were desalted on homemade SDB‐RPS (3m, 2241) stage tips and all samples were analyzed on the EASY‐nLC 1200 system interfaced online with the Q Exactive HF‐X mass spectrometer (Thermo). Peptides were dissolved in 0.1% formic acid, loaded onto a C18 trap column (100 µm × 20 mm, 3 µm particle size, 120 Å pore size) through auto‐sampler and then eluted into a C18 analytical column (75 µm × 250 mm, 2 µm particle size, 100 Å pore size). Mobile phase A (0.1% formic acid) and mobile phase B (90% ACN, 0.1% formic acid) were used to establish a 60 min separation gradient. A constant flow rate was set at 300 nL min^−1^. Data were acquired using a spray voltage of 2 kV, Ion funnel RF of 40, and ion transfer tube temperature of 320 °C. Each scan cycle consisted of one full‐scan mass spectrum (Res. 60 K, scan range 350–1500 m/z, AGC 300%, IT 20 ms) followed by MS/MS events (Res. 15 K, AGC 100%, IT auto). Cycle time was set to 2 s. The isolation window was set at 1.6 Da. Dynamic exclusion time was set to 35 s. The normalized collision energy was set at 30%. Raw files were searched with the MaxQuant (version 1.6.6) against the Swissprot database. A false discovery rate cutoff of 1% was applied at the peptide spectrum match (PSM) and protein levels. Proteins were quantified using the label‐free quantification (LFQ) algorithm.

### Statistical Analysis

Densitometry was performed using ImageJ software for the quantitative analysis of the bands on the western blots. Electrophysiological data were analyzed offline with Qstudio developed by Dr. Feng Qin at State University of New York at Buffalo, Clampfit (Molecular Devices, Sunnyvale, CA), IGOR (Wavemetrics, Lake Oswego, OR), SigmaPlot (SPSS Science, Chicago, IL), and OriginPro (OriginLab Corporation, MA). For concentration response analysis, the modified Hill equation was used: Y = A1 + (A2 – A1)/[1 + 10 ^ (logEC_50_ – X) * *n*_H_], in which EC_50_ is the half maximal effective concentration, and *n*_H_ is the Hill coefficient. The summary data are presented as mean ± standard deviation (S.D.) as indicated, from a population of cells (*n*). Statistical tests of significance were performed by Student's *t*‐test for one‐group comparison and two‐group comparison or one‐way analysis of variance (ANOVA) with Bonferroni's post‐tests for multiple group comparisons. For animal survival analysis, the Kaplan–Meier method was adopted to generate graphs, and the survival curves were analyzed with log‐rank analysis. Significant difference is indicated by a *p* value less than 0.05 (*p < 0.05, ** *p* < 0.01, *** *p* < 0.001).

## Conflict of Interest

The authors declare no conflict of interest.

## Authors Contribution

Y.Y.G. and Y.G. contributed equally to this work. Z.D.Z. and J.Y. designed and supervised the study. Y.G., Y.G., Y.H., Y.Z., Y.L., Y.W., D.J., Y.Z., B.Z., and J.Y. carried out the experiments and analyzed data. C.X. and Z.L. provided technical support and suggestions. Y.G., Y.G., J.Y., and B.Z. wrote the paper with inputs from all other authors. The authors read and approved the final manuscript.

## Supporting information

Supporting Information

Supplemental Table 1

Supplemental Table 2

Supplemental Table 3

Supplemental Video 1

Supplemental Video 2

Supplemental Video 3

Supplemental Video 4

Supplemental Video 5

Supplemental Video 6

## Data Availability

The data that support the findings of this study are available from the corresponding author upon reasonable request.
