# Supplementary material for: The Transient Receptor Potential Vanilloid 2 (TRPV2) Channel Facilitates Virus Infection Through the Ca2+‐LRMDA Axis in Myeloid Cells
Source: Adv Sci (Weinh). 2022 Oct 19;9(34):2202857. doi: 10.1002/advs.202202857 (PMC9731701; doi:10.1002/advs.202202857)
Supplement: Supplementary file 1 — Supporting Information [file ADVS-9-2202857-s007.pdf]

Figure S1

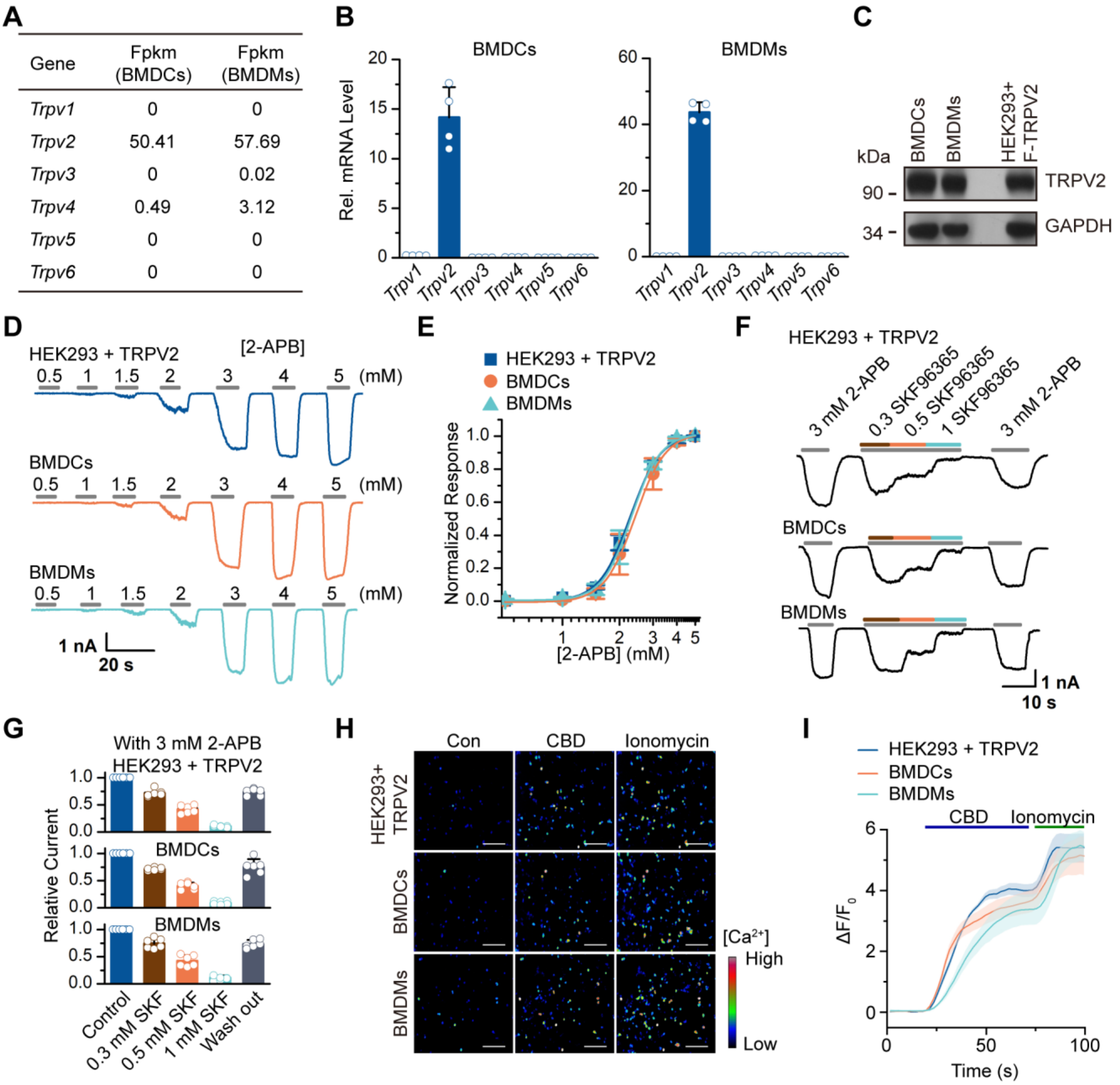

Gene	Fpkm (BMDCs)	Fpkm (BMDMs)
Trpv1	0	0
Trpv2	50.41	57.69
Trpv3	0	0.02
Trpv4	0.49	3.12
Trpv5	0	0
Trpv6	0	0

Figure S1 Functional TRPV2 is intensively expressed in BMDCs and BMDMs.
(A) The FPKM values of *Trpv* genes in BMDCs and BMDMs.
(B) qRT-PCR analysis of mRNA levels of *Trpv* genes in BMDCs and BMDMs.
(C) Immunoblot analysis (with anti-TRPV2 and GAPDH) in BMDCs, BMDMs and HEK293 cells transfected with FLAG-TRPV2.
(D) A representative whole-cell recording of HEK293 cells transfected with TRPV2 (upper), BMDCs (middle) and BMDMs (lower) that were stimulated with 2-APB (0.5-5 mM) in neutral condition with the holding potential of –60 mV.
(E) Dose–response curves for 2-APB-evoked currents in HEK293 cells transfected with TRPV2, BMDCs and BMDMs (n = 5).
(F, G) A representative whole-cell recording (F) and summary of relative currents (G, n = 6) of HEK293 cells transfected with TRPV2 (upper), BMDCs (middle) and BMDMs (lower) that were stimulated with 2-APB (3 mM) with or without SKF96365 (0.3-1 mM) followed by wash out and restimulation of 2-APB (3 mM) with the holding potential of –60 mV.
(H) Fluorescent microscopy analysis of Ca^{2+} imaging in HEK293 cells transfected with TRPV2 (upper), BMDCs (middle) and BMDMs (lower) that were transfected with GCaMP6m and consecutively challenged with CBD (30 µM) followed by ionomycin (1 µM). The colored bar indicated relative calcium levels.
(I) Averaged responses of HEK293 cells transfected with TRPV2, BMDCs and BMDMs that were treated as in (H). GCaMP6m fluorescence changes were computed as $(F_i – F_0)/F_0$, where F_i represented fluorescence intensity at any frame and F_0 was the baseline fluorescence calculated from the averaged fluorescence of the first 10 frames.
Graphs show mean ± S.D. in (B, E, G, I). Scale bars represent 200 µm in (H). Data are representative of two (B-I) independent experiments.

Figure S2

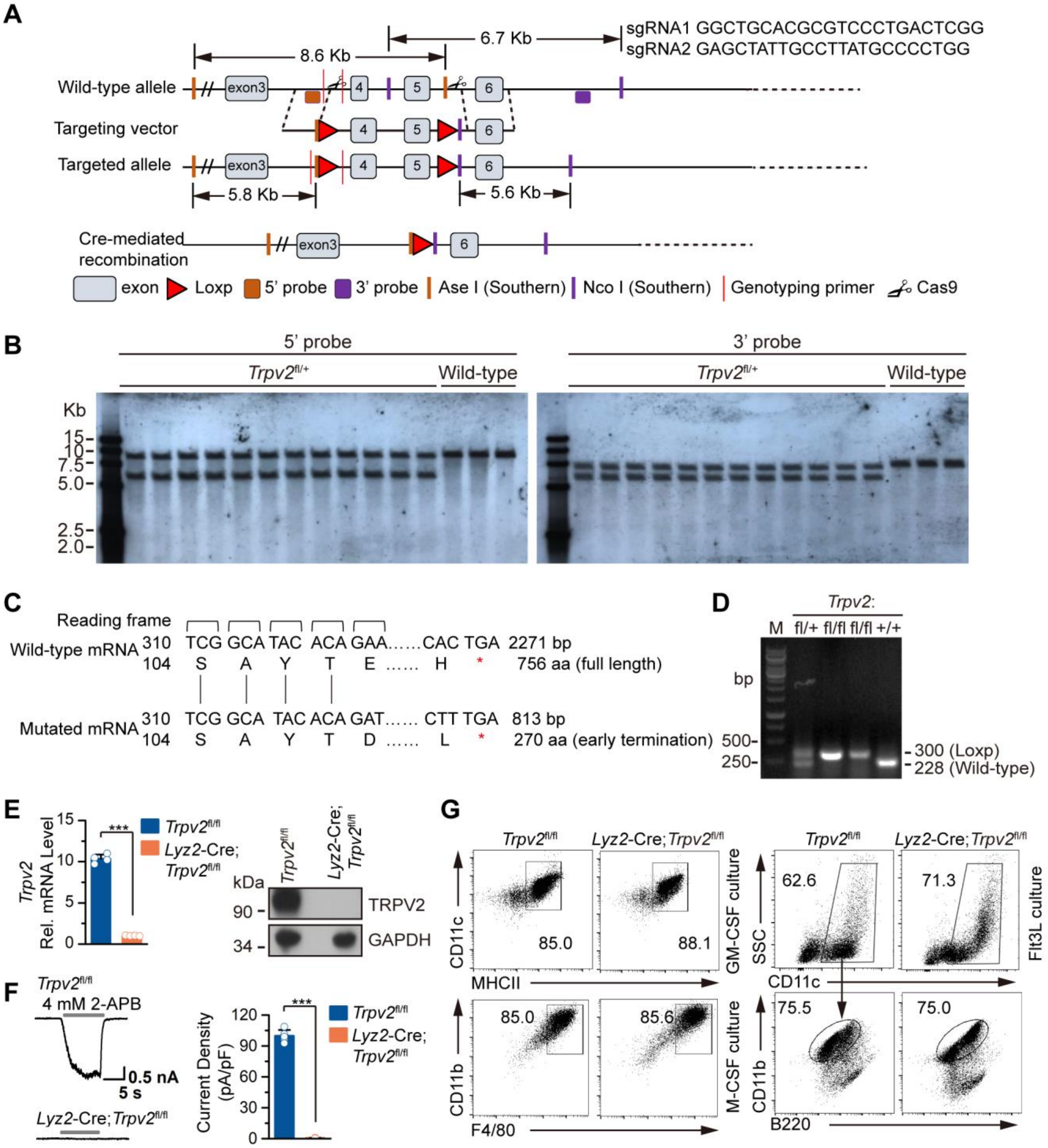

Figure S2 Generation of *Trpv2*$^{fl/fl}$ mice.

(A) A scheme for CRISPR/Cas9-mediated genome editing of the *Trpv2* gene locus.

(B) Southern blot analysis of the F1 *Trpv2*$^{fl/+}$ and wild-type C57BL/6 mice.

(C) Gene sequence and reading frame of wild-type and mutated *Trpv2* alleles.

(D) Genotyping analysis of tail DNAs from *Trpv2*$^{fl/fl}$, *Trpv2*$^{fl/+}$ and *Trpv2*$^{+/+}$ mice.

(E) qRT-PCR of *Trpv2* mRNA (left) and immunoblot analysis of TRPV2 protein (right) in *Trpv2*$^{fl/fl}$ and *Lyz2*-Cre;*Trpv2*$^{fl/fl}$ BMDMs.

(F) A representative whole-cell recording of *Trpv2*$^{fl/fl}$ (left, upper) and *Lyz2*-Cre;*Trpv2*$^{fl/fl}$ (left, lower) BMDMs. The cell was exposed to 4 mM 2-APB in neutral condition (pH 7.4). Summary data (right, n=5) of current densities evoked by 4 mM 2-APB at a holding potential of −60 mV.

(G) Flow cytometry analysis of in vitro differentiated *Trpv2*$^{fl/fl}$ and *Lyz2*-Cre *Trpv2*$^{fl/fl}$ BMDCs, BMDMs and cDCs in the presence of GM-CSF, M-CSF and Flt3L, respectively.

****P*<0.001 (two-tailed student's *t*-test in E-F). Graphs show mean ± S.D. in (E-F). Data are representative of two (E-G) independent experiments.

Figure S3

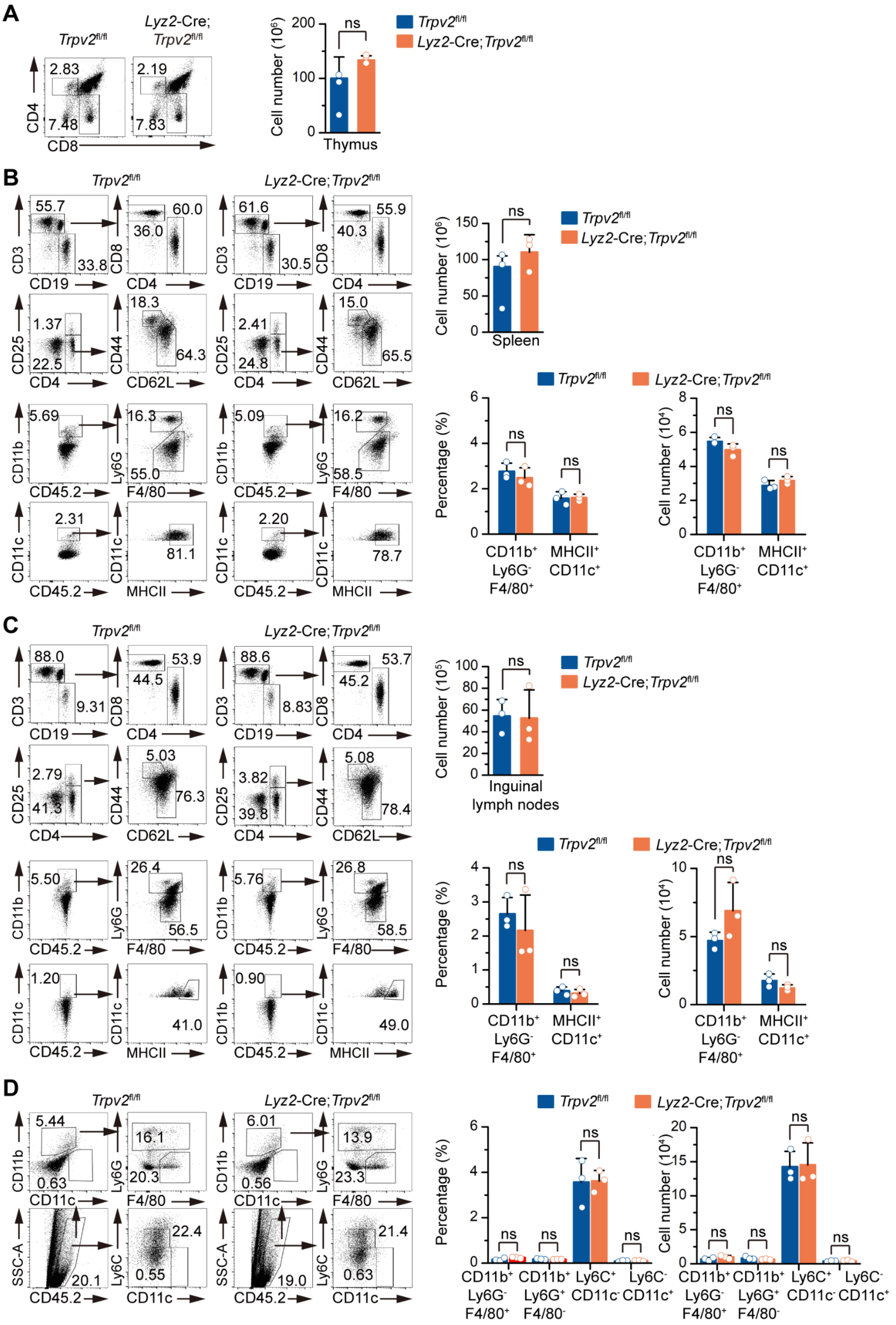

Figure S3 Knockout of TRPV2 in myeloid cells does not affect the homeostasis of immune cells.

(A-C) Flow cytometry and quantitative analyses of various immune cells in thymus (A), spleen (B) and inguinal lymph nodes (C) from *Trpv2*$^{fl/fl}$ and *Lyz2*-Cre;*Trpv2*$^{fl/fl}$ (n=3) mice.

(D) Flow cytometry analysis of myeloid or lymphoid cells in the peripheral blood from *Trpv2*$^{fl/fl}$ and *Lyz2*-Cre;*Trpv2*$^{fl/fl}$ (n=3) mice.

ns, not significant (two-tailed student's *t*-test in A-D). Graphs show mean ±S.D. (A-D). Data are representative of two independent experiments (A-D).

Figure S4

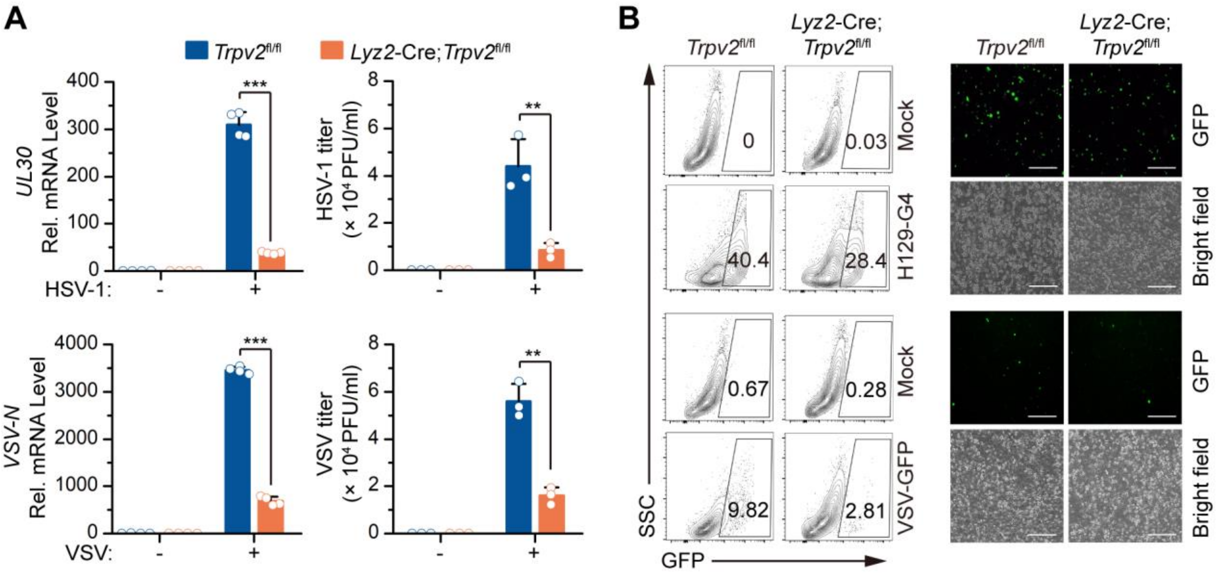

Figure S4 Knockout of TRPV2 inhibits HSV-1 and VSV infection in BMDMs.

(A) qRT-PCR analysis of HSV-1 *UL30* gene or VSV *N* gene in (left two graphs) and plaque assays of HSV-1 and VSV titers in the supernatants of (right two graphs) *Trpv2*$^{fl/fl}$ and *Lyz2*-Cre;*Trpv2*$^{fl/fl}$ BMDMs infected with HSV-1 or VSV for 12 hours.

(B) Flow cytometric analysis (left) and fluorescent microscopy imaging (right) of GFP signals in *Trpv2*$^{fl/fl}$ and *Lyz2*-Cre;*Trpv2*$^{fl/fl}$ BMDMs that were uninfected or infected with H129-G4 or VSV-GFP for 1 h followed by PBS wash twice and cultured in full medium for 12 h. Numbers adjacent to the outlined areas indicate percentages of GFP^{+} cells.

***P*<0.01; ****P*<0.001 (two-tailed student's *t*-test in A). Graphs show mean ± S.D. in (A). Scale bars represent 200 μm. Data are representative of two independent experiments.

Figure S5

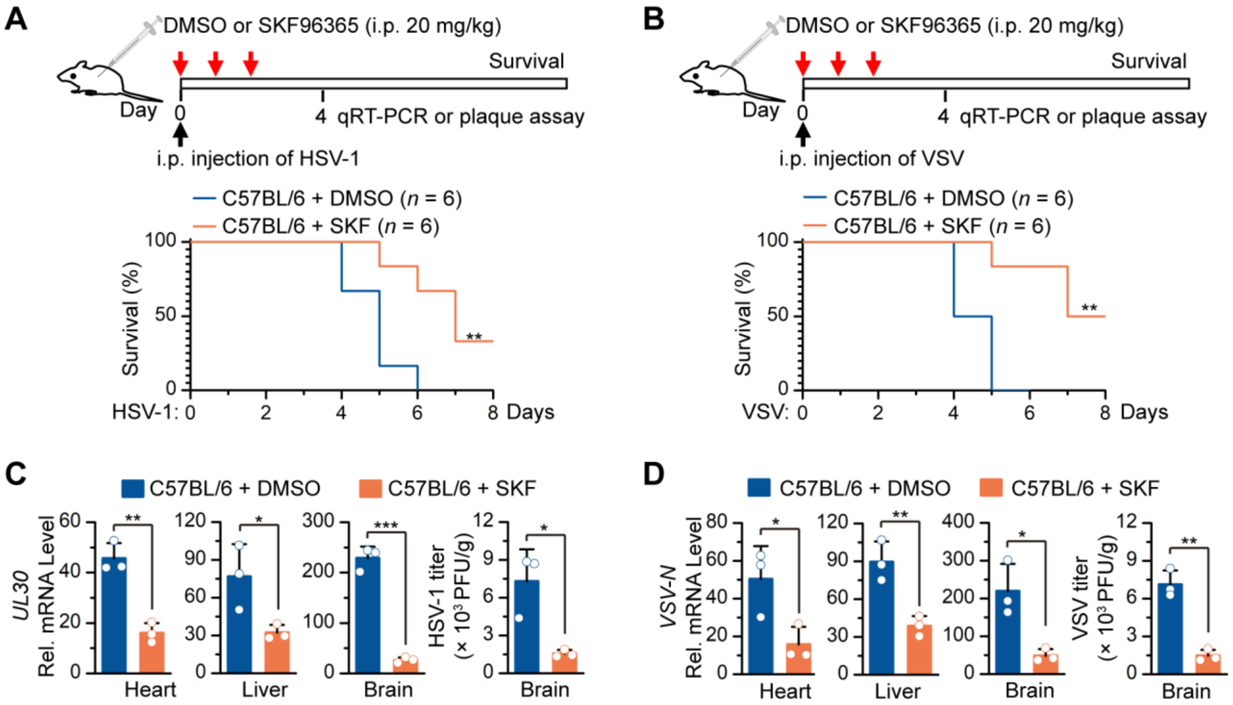

Figure S5 Inhibition of TRPV2 by SKF96365 leads to hyper-resistance to lethal HSV-1 and VSV infection in mice.

(A-B) A scheme of SKF96365 treatment and viral infection (upper) and survival (Kaplan–Meier curve) (lower) of wild-type C57BL/6 mice that were intraperitoneally injected with HSV-1 (2.5 x 10^6 PFU per mouse, A) or VSV (1 x 10^7 PFU per mouse, B) followed by intraperitoneal injection of DMSO (n=6) or SKF96365 (n=6) (20 mg/kg per mouse) for 3 successive days.

(C) qRT-PCR analysis of HSV-1 *UL30* gene (in the heart, liver, and brain) and HSV-1 titers (in the brain) from the wild-type C57BL/6 mice treated as in (A, left) (n=3) followed by intraperitoneal injection with HSV-1 (2.5 x 10^6 PFU per mouse) for 4 days.

(D) qRT-PCR analysis of VSV *N* gene (in the heart, liver, and brain) and VSV titers (in the heart and brain) from the wild-type C57BL/6 mice treated as in (B, right) (n=3) followed by intraperitoneal injection with VSV (1 x 10^7 PFU per mouse) for 4 days.

*P<0.05; **P<0.01; ***P<0.001 (two-tailed student's t-test or log-rank analysis). Graphs show mean ± S.D. in (A-D). Data are representative of two (A-D) independent experiments.

Figure S6

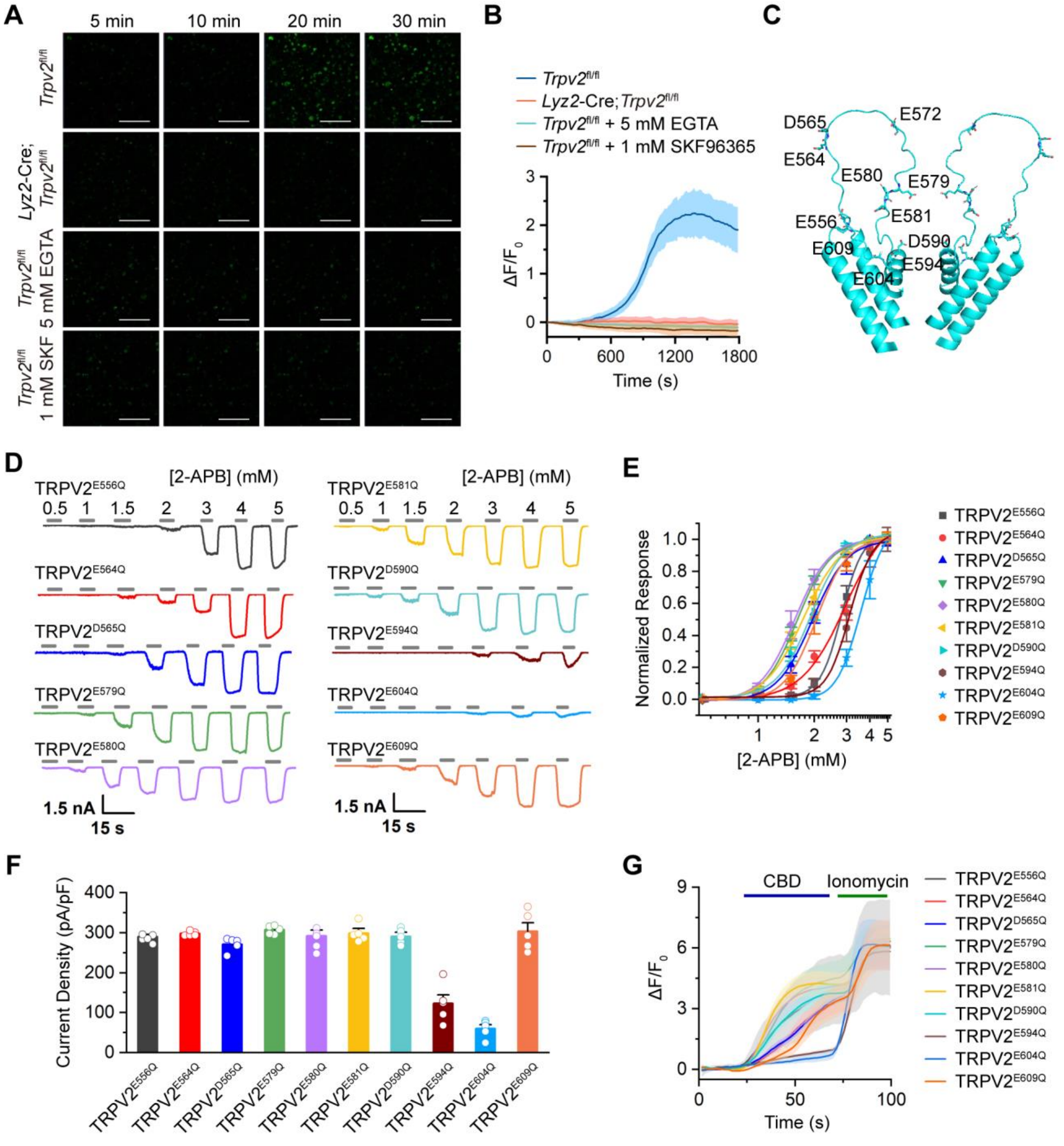

Figure S6 E572 of TRPV2 is required for its Ca^{2+} permeability.

(A-B) Fluorescent microscopy analysis of Ca^{2+} imaging (A) and quantitative analysis of the GFP signals (B) in *Trpv2*$^{fl/fl}$ and *Lyz2*-Cre;*Trpv2*$^{fl/fl}$ BMDMs infected with HSV-1 in the presence of EGTA (5 mM) or SKF96365 (1 mM).

(C) Acidic amino acid residues in the predicted TRPV2 S5-S6 structure from alfafold website.

(D) A representative whole-cell recording of HEK293 cells transfected with TRPV2^{E556Q}, TRPV2^{E564Q}, TRPV2^{D565Q}, TRPV2^{E579Q}, TRPV2^{E580Q}, TRPV2^{E581Q}, TRPV2^{D590Q}, TRPV2^{E594Q}, TRPV2^{E604Q} or TRPV2^{E609Q} that were stimulated with 2-APB (0.5-5 mM) with the holding potential of –60 mV.

(E) Dose–response curves for 2-APB-evoked currents in HEK293 cells transfected with TRPV2^{E556Q}, TRPV2^{E564Q}, TRPV2^{D565Q}, TRPV2^{E579Q}, TRPV2^{E580Q}, TRPV2^{E581Q}, TRPV2^{D590Q}, TRPV2^{E594Q}, TRPV2^{E604Q} or TRPV2^{E609Q}. Solid lines indicated fits with the Hill equation, which yielded EC_{50} = 2.81 ± 0.04 mM, n_H = 5.37 ± 0.85 for TRPV^{E556Q} (n = 5); EC_{50} = 2.98 ± 0.33 mM, n_H = 3.59 ± 1.05 for TRPV^{E564Q} (n = 5); EC_{50} = 1.95 ± 0.06 mM, n_H = 4.61 ± 0.63 for TRPV2^{D565Q} (n = 5); EC_{50} = 2.26 ± 0.03 mM, n_H = 5.39 ± 0.31 for TRPV^{E579Q} (n = 5); EC_{50} = 1.57 ± 0.03 mM, n_H = 4.67 ± 0.43 for TRPV2^{E580Q} (n = 5); EC_{50} = 1.75 ± 0.03 mM, n_H = 4.19 ± 0.36 for TRPV2^{E581Q} (n = 5); EC_{50} = 1.91 ± 0.06 mM, n_H = 4.75 ± 0.71 for TRPV2^{D590Q} (n = 5); EC_{50} = 3.12 ± 0.06 mM, n_H = 6.91 ± 1.12 for TRPV^{E594Q} (n = 5); EC_{50} = 3.58 ± 0.01 mM, n_H = 6.65 ± 0.11 for TRPV^{E604Q} (n = 5); EC_{50} = 2.05 ± 0.07 mM, n_H = 5.12 ± 0.85 for TRPV2^{E609Q} (n = 5).

(F) Summary of relative currents elicited by 5 mM 2-APB in HEK293 cells (n=5) transfected with TRPV2^{E556Q}, TRPV2^{E564Q}, TRPV2^{D565Q}, TRPV2^{E579Q}, TRPV2^{E580Q}, TRPV2^{E581Q}, TRPV2^{D590Q}, TRPV2^{E594Q}, TRPV2^{E604Q} or TRPV2^{E609Q}.

(G) Averaged responses of HEK 293T cells transfected with GCaMP6m and TRPV2^{E556Q}, TRPV2^{E564Q}, TRPV2^{D565Q}, TRPV2^{E579Q}, TRPV2^{E580Q}, TRPV2^{E581Q}, TRPV2^{D590Q}, TRPV2^{E594Q}, TRPV2^{E604Q} or TRPV2^{E609Q} that were consecutively stimulated with CBD (30 μM) followed by ionomycin (1 μM) stimulation. GCaMP6m fluorescence changes were computed as $(F_i–F_0)/F_0$, where F_i represented fluorescence intensity at any frame and F_0 was the baseline fluorescence calculated from the averaged fluorescence of the first 10 frames.

Graphs show mean ± S.D. in (B, E-G). Scale bars represent 100 μm in (A). Data are representative of two independent experiments (A-B, D-G).

Figure S7

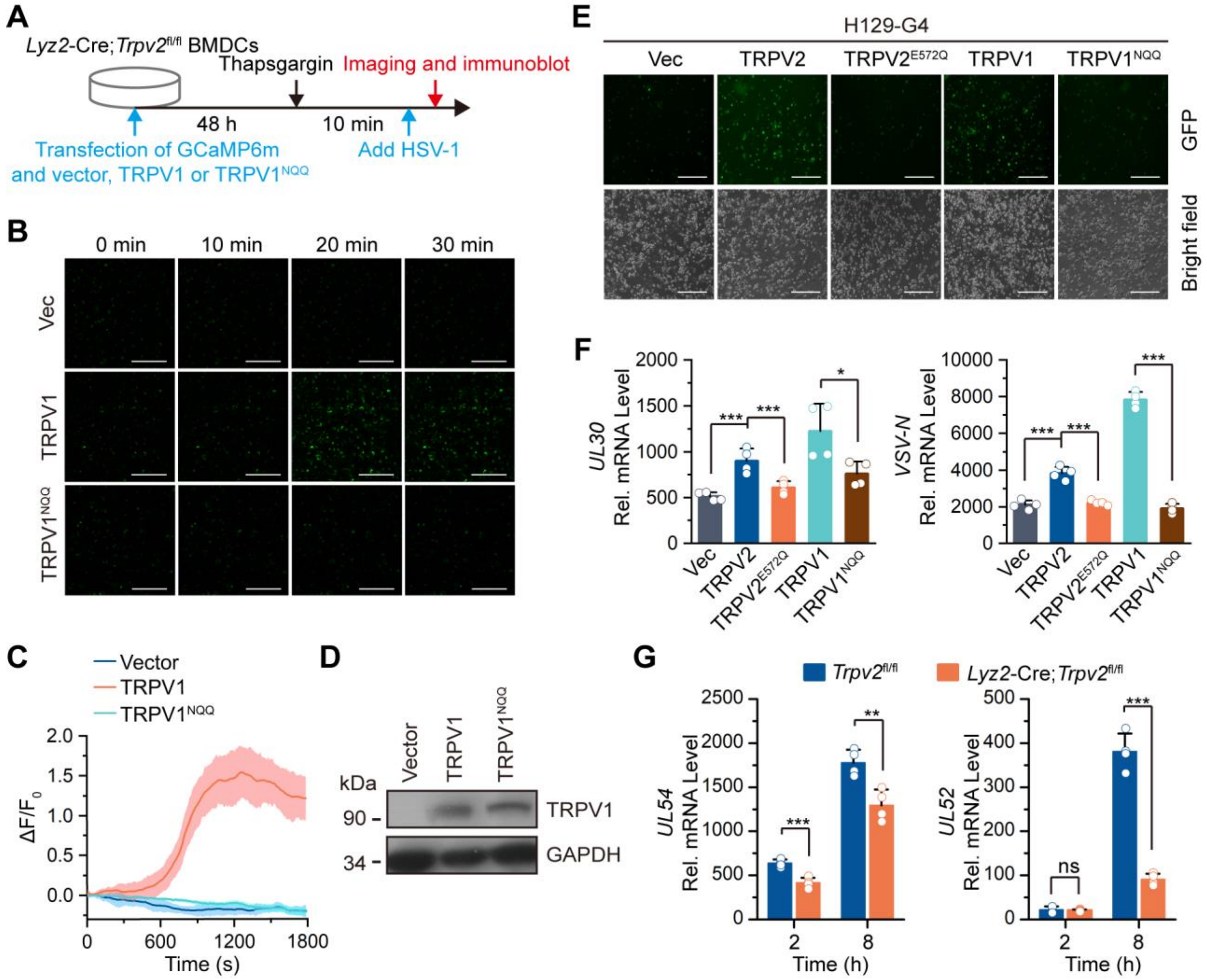

Figure S7 Reconstitution of TRPV1 restores viral infection in *Lyz2*-Cre;*Trpv2*$^{fl/fl}$ BMDCs.

(A) A scheme of experiments for (B) and (C).

(B-C) Fluorescent microscopy analysis of Ca^{2+} imaging (B) and quantitative analysis of the GFP signals (C) in *Lyz2*-Cre;*Trpv2*$^{fl/fl}$ BMDCs transfected with GCaMP6m and wild-type TRPV1 or TRPV1$^{D646N/E648/651Q}$ followed by HSV-1 infection.

(D) Immunoblot analysis of TRPV1 or TRPV1$^{D646N/E648/651Q}$ in *Lyz2*-Cre;*Trpv2*$^{fl/fl}$ BMDCs that were used for (E-F).

(E) Fluorescent microscopy imaging of *Lyz2*-Cre;*Trpv2*$^{fl/fl}$ BMDCs that were transfected with an empty vector, wild-type TRPV2, TRPV2^{E572Q}, wild-type TRPV1 or TRPV1$^{D646N/E648/651Q}$ followed by infection with H129-G4 for 12 h.

(F) qRT-PCR analysis of HSV-1 *UL30* gene (left) or VSV *N* gene (right) in *Lyz2*-Cre;*Trpv2*$^{fl/fl}$ BMDCs that were transfected with an empty vector, TRPV2, TRPV2^{E572Q}, TRPV1 or TRPV1$^{D646N/E648/651Q}$ infected with HSV-1 or VSV for 12 h.

(G) qRT-PCR analysis of HSV-1 *UL54* (immediate early gene) and *UL52* (late expressed gene) genes in *Trpv2*$^{fl/fl}$ and *Lyz2*-Cre;*Trpv2*$^{fl/fl}$ BMDCs (n = 4) that were infected with HSV-1 for 2 or 8 h.

*P<0.05; **P<0.01; ***P<0.001; ns, not significant (two-tailed student's *t*-test in F, G). Graphs show mean ± S.D. in (C, F-G). Scale bars represent 100 μm (B) or 200 μm (E). Data are representative of two independent experiments (B-G).

Figure S8

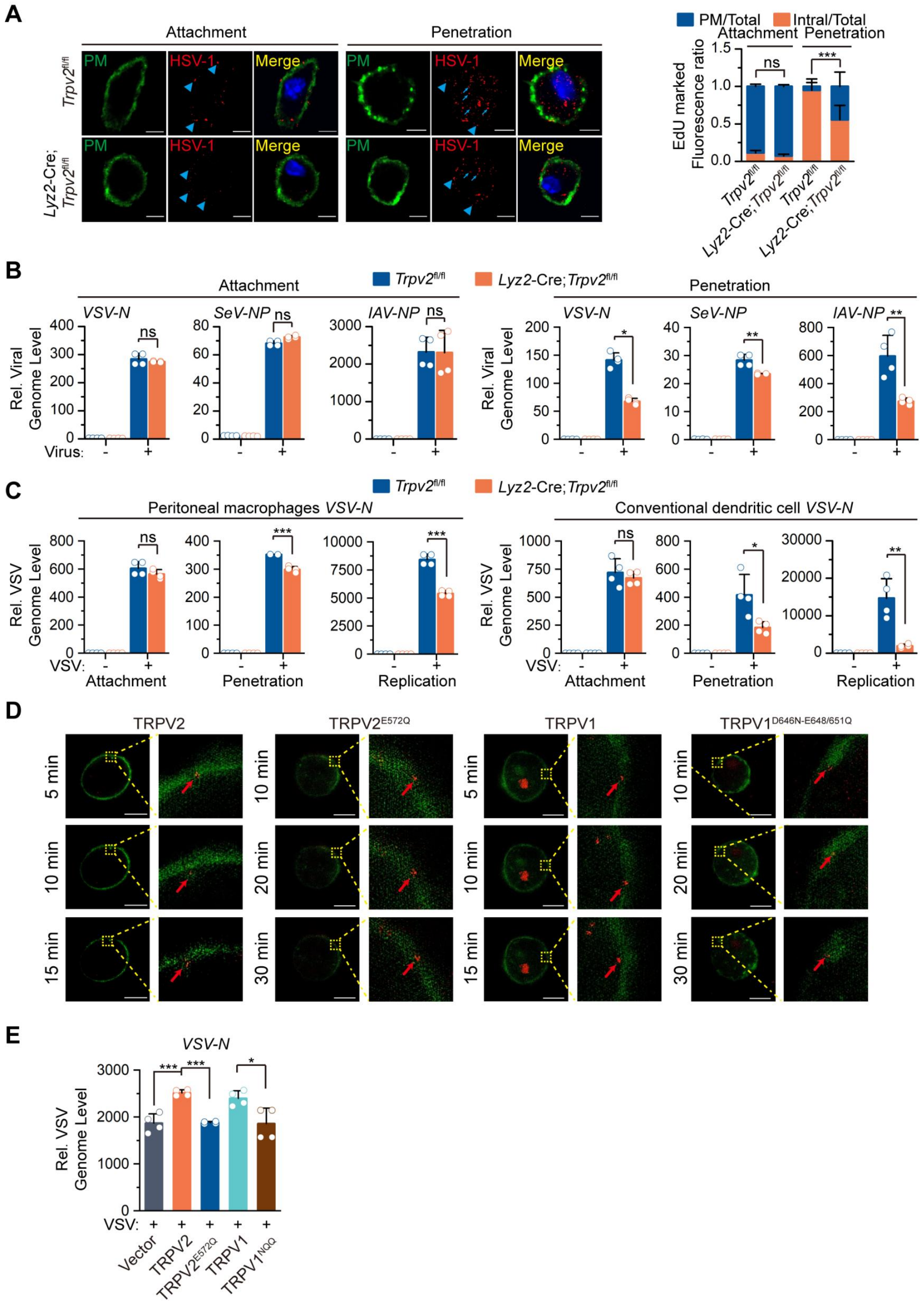

A
Attachment
Penetration
PM
HSV-1
Merge
Trpv2fl/fl
Lyz2-Cre; Trpv2fl/fl
PM/Total
Intral/Total
EdU marked Fluorescence ratio
ns
***
B
Attachment
Penetration
Trpv2fl/fl
Lyz2-Cre;Trpv2fl/fl
VSV-N
SeV-NP
IAV-NP
Rel. Viral Genome Level
Virus:
C
Peritoneal macrophages VSV-N
Conventional dendritic cell VSV-N
Rel. VSV Genome Level
VSV:
Attachment
Penetration
Replication
D
TRPV2
TRPV2E572Q
TRPV1
TRPV1D646N-E648/651Q
5 min
10 min
15 min
20 min
30 min
E
VSV-N
Rel. VSV Genome Level
Vector
TRPV2
TRPV2E572Q
TRPV1
TRPV1NQQ

Figure S8 TRPV2 deficiency inhibits the penetration of viruses in myeloid cells.

(A) Fluorescent microscopy imaging (left) and quantification analysis (right) of the EdU signals in*Trpv2*$^{fl/fl}$ and *Lyz2*-Cre;*Trpv2*$^{fl/fl}$ BMDCs that were incubated with EdU-labeled HSV-1 at 4 ^{o}C for 1 h followed by twice PBS wash (attachment) or culture at 37 ^{o}C for 1 h (penetration). The cells were stained with CellMask^{TM} green (green) and Apollo reaction cocktail (red) before subject to fluorescent microscopy assays. Arrowheads indicated the EdU signals on the cell membrane. Arrows indicated EdU signals in the cytosol. PM, plasma membrane ; Intra, intracellular.

(B) qRT-PCR analysis of VSV, SeV or PR8 IAV genome of the attached and the penetrated viruses in *Trpv2*$^{fl/fl}$ and *Lyz2*-Cre *Trpv2*$^{fl/fl}$ BMDCs.

(C) qRT-PCR analysis of VSV genome of the attached, penetrated and replicated VSV in peritoneal macrophages (left) or cDCs (right) that were infected with VSV at 4 ^{o}C for 1 h followed by twice PBS wash (attachment), followed by culture at 37 ^{o}C for 1 h (penetration), or culture at 37 ^{o}C for 12 h (replication).

(D) Representative images captured from the a movie recording *Lyz2*-Cre;*Trpv2*$^{fl/fl}$ BMDCs that were transfected with wild-type TRPV2, TRPV2^{E572Q}, wild-type TRPV1 or TRPV1$^{D646N/E648/651Q}$ followed by infection with Near-infrared quantum dots encapsulated in the SV40 virus-like particles. Arrows indicated the SV40 virus-like particles.

(E) qRT-PCR analysis of VSV genome of the penetrated VSV in *Lyz2*-Cre;*Trpv2*$^{fl/fl}$ BMDCs that were transfected with an empty vector, wild-type TRPV2, TRPV2^{E572Q}, wild-type TRPV1 or TRPV1$^{D646N/E648/651Q}$ that were infected with VSV at 4 ^{o}C for 1 h followed by twice PBS wash and culture at 37 ^{o}C for 1 h.

**P*<0.05; ***P*<0.01; ****P*<0.001; ns, not significant (two-tailed student's *t*-test in A-C, E). Graphs show mean ± S.D. in (A-C, E). Scale bars represent 5 μm in (A, D). Data are representative of two independent experiments (A-E).

Figure S9

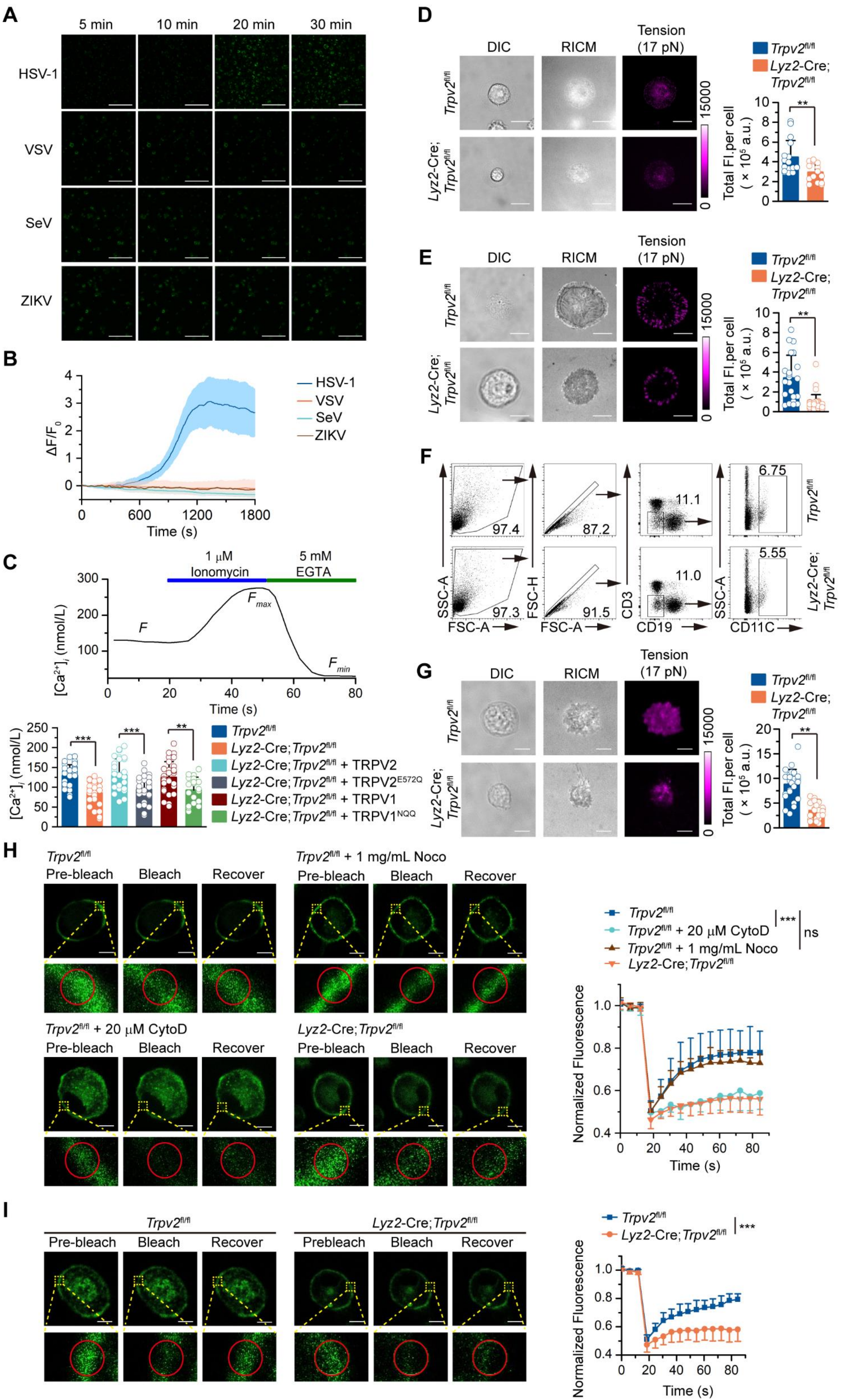

A
5 min
10 min
20 min
30 min
HSV-1
VSV
SeV
ZIKV
B
ΔF/F0
Time (s)
HSV-1
VSV
SeV
ZIKV
C
1 μM Ionomycin
5 mM EGTA
F
Fmax
Fmin
[Ca2+]i (nmol/L)
Time (s)
Trpv2fl/fl
Lyz2-Cre;Trpv2fl/fl
Lyz2-Cre;Trpv2fl/fl + TRPV2
Lyz2-Cre;Trpv2fl/fl + TRPV2E572Q
Lyz2-Cre;Trpv2fl/fl + TRPV1
Lyz2-Cre;Trpv2fl/fl + TRPV1NQQ
D
DIC
RICM
Tension (17 pN)
Trpv2fl/fl
Lyz2-Cre; Trpv2fl/fl
Total FI. per cell (× 10^5 a.u.)
E
F
97.4
87.2
11.1
6.75
97.3
91.5
11.0
5.55
SSC-A
FSC-A
FSC-H
CD3
CD19
CD11C
G
H
Trpv2fl/fl + 1 mg/mL Noco
Trpv2fl/fl + 20 μM CytoD
Pre-bleach
Bleach
Recover
Normalized Fluorescence
ns
I
Prebleach

Figure S9 Knockout of TRPV2 in myeloid cells impairs the tension and mobility of cell membrane.

(A-B) Fluorescent microscopy analysis of Ca^{2+} imaging (A) and quantitative analysis of GFP signals (B) in wild-type BMDCs that were transfected with GCaMP6m followed by infection with HSV-1, VSV, SeV or ZIKV.

(C) Example of intracellular calcium concentration change curve for BMDCs based on Fluo-3 measurements (upper). Summary data of intracellular calcium concentration of *Trpv2*$^{fl/fl}$ and *Lyz2*-Cre;*Trpv2*$^{fl/fl}$ BMDCs and *Lyz2*-Cre;*Trpv2*$^{fl/fl}$ BMDCs that were transfected with TRPV2, TRPV2^{E572Q}, TRPV1 or TRPV1^{NQQ} (lower).

(D-E) Representative images of DIC, RICM and TIRF microscopy (left) and statistic total fluorescent intensities (right) of *Trpv2*$^{fl/fl}$ and *Lyz2*-Cre;*Trpv2*$^{fl/fl}$ Flt3L-cDCs (D) or peritoneal macrophages (E) that were seeded on a 17 pN DNA tension probe.

(F) Flow cytometry sorting of cDCs from spleen of *Trpv2*$^{fl/fl}$ and *Lyz2*-Cre;*Trpv2*$^{fl/fl}$ mice.

(G) Representative images of DIC, RICM and TIRF images (left) and statistic total fluorescent intensities (right) of *Trpv2*$^{fl/fl}$ and *Lyz2*-Cre;*Trpv2*$^{fl/fl}$ splenic cDCs that were seed on a 17 pN DNA tension probe.

(H) Representative images (left) and quantitative analysis (right) of FRAP in the cell membrane of *Trpv2*$^{fl/fl}$ and *Lyz2*-Cre;*Trpv2*$^{fl/fl}$ BMDCs treated with or without Noco (1 mg/mL) or CytoD (20 μM) for 30 minutes.

(I) Representative images (left) and quantitative analysis (right) of FRAP in the cell membranes of *Trpv2*$^{fl/fl}$ and *Lyz2*-Cre;*Trpv2*$^{fl/fl}$ BMDMs.

*$P<0.05$; **$P<0.01$; ***$P<0.001$; ns, not significant (two-tailed student's *t*-test in C-E, G-I). Graphs show mean ± S.D. in (B-E, G-I). Scale bars represent 100 μm (A), 15 μm (D), 10 μm (E) or 5 μm (G-I). Data are representative of two independent experiments (A-I).

Figure S10

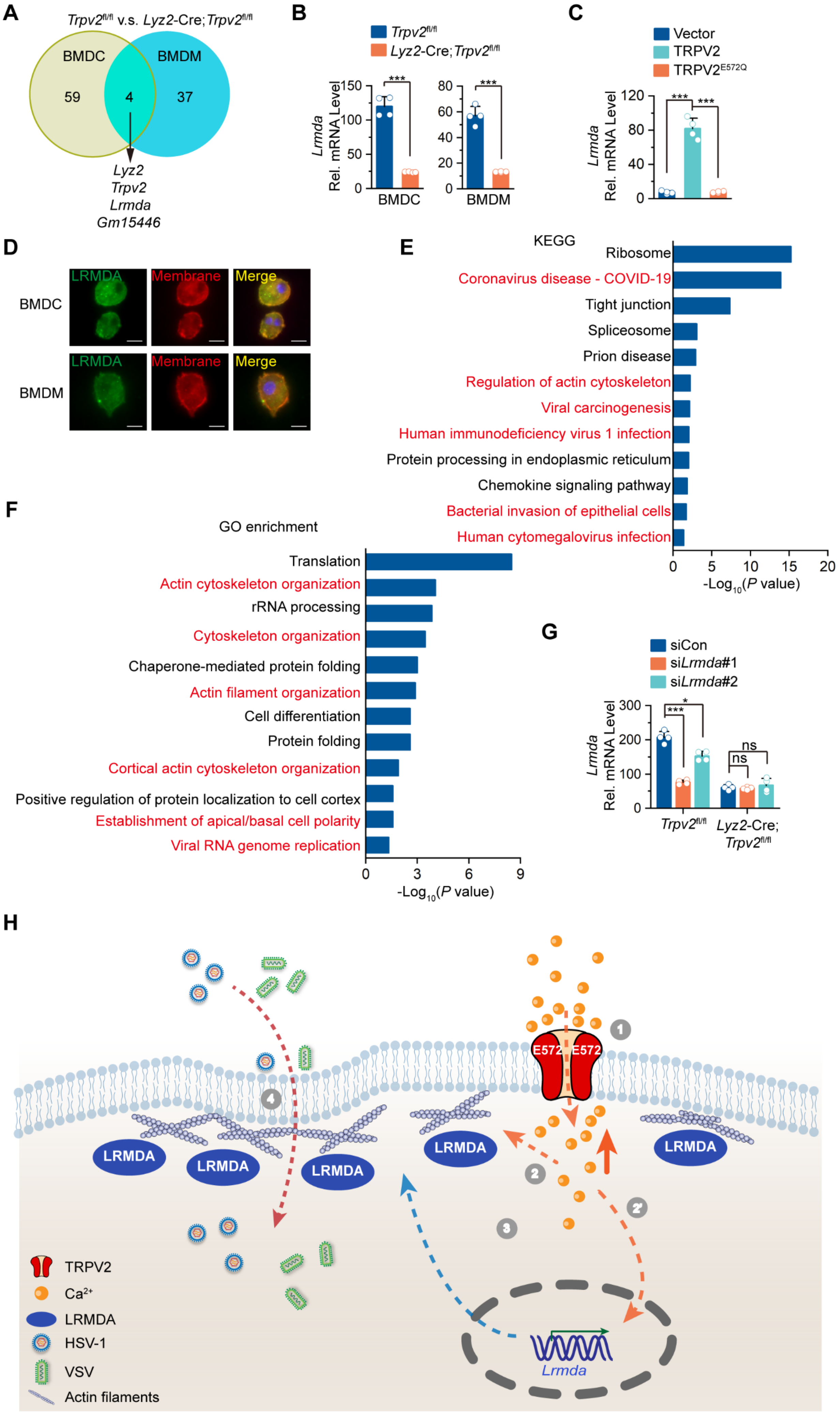
A
Trpv2fl/fl v.s. Lyz2-Cre;Trpv2fl/fl
BMDC
BMDM
59
4
37
Lyz2
Trpv2
Lrmda
Gm15446
B
Trpv2fl/fl
Lyz2-Cre;Trpv2fl/fl
Lrmda Rel. mRNA Level
***
BMDC
BMDM
C
Vector
TRPV2
TRPV2E572Q
Lrmda Rel. mRNA Level
D
LRMDA
Membrane
Merge
BMDC
BMDM
E
KEGG
Ribosome
Coronavirus disease - COVID-19
Tight junction
Spliceosome
Prion disease
Regulation of actin cytoskeleton
Viral carcinogenesis
Human immunodeficiency virus 1 infection
Protein processing in endoplasmic reticulum
Chemokine signaling pathway
Bacterial invasion of epithelial cells
Human cytomegalovirus infection
-Log10(P value)
F
GO enrichment
Translation
Actin cytoskeleton organization
rRNA processing
Cytoskeleton organization
Chaperone-mediated protein folding
Actin filament organization
Cell differentiation
Protein folding
Cortical actin cytoskeleton organization
Positive regulation of protein localization to cell cortex
Establishment of apical/basal cell polarity
Viral RNA genome replication
-Log10(P value)
G
siCon
siLrmda#1
siLrmda#2
Lrmda Rel. mRNA Level
***
*
ns
ns
Trpv2fl/fl
Lyz2-Cre; Trpv2fl/fl
H
E572
E572
LRMDA
Lrmda
TRPV2
Ca2+
LRMDA
HSV-1
VSV
Actin filaments

Figure S10 Knockout of TRPV2 downregulates LRMDA in myeloid cells.

(A) Differentially expressed gene in *Trpv2*$^{fl/fl}$ and *Lyz2*-Cre;*Trpv2*$^{fl/fl}$ BMDCs or BMDMs. Adjusted $P \leq 0.05$ and cut-off values of $\log_2$[Fold Change] ≥ 1 or ≤ -1. Numbers indicated genes that were differentially expressed in *Trpv2*$^{fl/fl}$ and *Lyz2*-Cre;*Trpv2*$^{fl/fl}$ BMDCs or BMDMs.

(B) qRT-PCR analysis of *Lrmda* mRNA level in *Trpv2*$^{fl/fl}$ and *Lyz2*-Cre;*Trpv2*$^{fl/fl}$ BMDCs or BMDMs.

(C) qRT-PCR analysis of *Lrmda* mRNA in *Lyz2*-Cre;*Trpv2*$^{fl/fl}$ BMDCs that were transfected with an empty vector, TRPV2, or TRPV2^{E572Q} for 48 h.

(D) Representative images of TIRF microscopy of BMDCs and BMDMs transfected with LRMDA-GFP for 48 h followed by immunofluorescent staining with anti-Na^+/K^+ ATPase (red).

(E-F) KEGG (E) and GO (F) pathway enrichment of the potential LRMDA-interacting proteins obtained from LC/MS analysis.

(G) qRT-PCR analysis of *Lrmda* in *Trpv2*$^{fl/fl}$ and *Lyz2*-Cre;*Trpv2*$^{fl/fl}$ BMDCs that were transfected with siCon, si*Lrmda*#1 or si*Lrmda*#2.

(H) A model of TRPV2-mediated virus infection in myeloid cells. TRPV2 mediates homeostatic and virus-induced Ca^{2+} influx in myeloid cells. The Ca^{2+} regulates actin cytoskeleton remodeling to promote viral penetration. In addition, the Ca^{2+} supports upregulation of LRMDA which promotes the tension and mobility of cell membrane, thereby facilitating viral penetration and infections.

*$P<0.05$; **$P<0.01$; ***$P<0.001$; ns, not significant (two-tailed student's *t*-test in B-C, G). Graphs show mean ± S.D. in (B-C, G). Scale bars represent 5 μm (D). Data are representative of two independent experiments (B-C, G).
